# Effects of preferred music listening on physical and psychological parameters in sports: a systematic review and meta-analysis with meta-regression

**DOI:** 10.1186/s13102-025-01470-2

**Published:** 2025-12-22

**Authors:** Marc Niering, Benedikt Zirkel, Paul Munkelt, Franziska Gellert, Rainer Beurskens, Johanna Seifert, Alexander Glahn

**Affiliations:** 1 Institute of Biomechanics and Neurosciences, Nordic Science, Hannover, Germany; 2 School of Sports, Psychology and Education, Triagon Academy Munich, Ismaning, Germany; 3 Department of Sports Medicine and Health Promotion, Friedrich-Schiller- University Jena, Jena, Germany; 4 Exercise Science & Neuroscience Unit, Department of Exercise & Health, Paderborn University, Paderborn, Germany; 5 Department of Health and Social Affairs, FHM Bielefeld - University of Applied Sciences, Bielefeld, Germany; 6 Department of Psychiatry, Social Psychiatry, and Psychotherapy, Hannover Medical School, Carl-Neuberg Straße 1, Hannover, 30625 Germany

**Keywords:** Athletic performance, Motivation, Athletes, Physical exertion, Music preference

## Abstract

**Objective:**

This meta-analysis examined the effects of preferred music listening (PML) versus non-preferred music listening (NPML) and no music listening (NML) on psychological and physical performance outcomes in adolescent and adult athletes.

**Methods:**

A systematic literature search was conducted in Embase, PubMed, PsycInfo, and MEDLINE. After screening 3146 records and applying predefined eligibility criteria, 41 studies were included in the meta-analysis. Data were synthesized for psychological parameters (perceived exertion, motivation, affective response) and physical parameters (strength endurance, power output, maximal strength, aerobic endurance, speed). Meta-regression analyses were performed to identify potential moderating effects of age, sex, music choice, and timing.

**Results:**

Statistically significant overall effects favoring PML were found for psychological outcomes, including a reduction in perceived exertion (SMD = − 0.36, 95% CI [− 0.65, − 0.08]), an increase in motivation (SMD = 0.85, 95% CI [0.60, 1.10]), and a more positive affective response (SMD = 1.16, 95% CI [0.13, 2.20]). For physical outcomes, significant between-condition differences were observed for strength endurance (SMD = 0.72, 95% CI [0.42, 1.01]), maximal strength (SMD = 0.53, 95% CI [0.20, 0.85]), and power output (SMD = 0.47, 95% CI [0.12, 0.81]), however subgroup comparisons with NPML were less consistent due to high heterogeneity. NPML showed slightly higher values than NML in some psychological and physical parameters, while no advantage was observed for speed or aerobic endurance.

**Conclusion:**

PML was associated with higher motivation, more positive affective responses, lower perceived exertion, and superior strength- and power-related performance compared to non-preferred and no music conditions. These findings reflect between-condition comparisons and emphasize the importance of individual preference in optimizing exercise experiences.

**Trial registration:**

PROSPERO registration number: CRD420251083551.

**Supplementary Information:**

The online version contains supplementary material available at 10.1186/s13102-025-01470-2.

## Key points

• PML is associated with higher motivation, more positive affective responses, and lower perceived exertion compared to NML.

• PML improves strength endurance, maximal strength, and power output, showing clear benefits for physical performance.

• NPML is superior to NML for motivation, but shows no advantage for speed.

**Supplementary Information:**

The online version contains supplementary material available at 10.1186/s13102-025-01470-2.

## Introduction

Listening to music elicits measurable physical, psychological, and psychophysiological responses across various contexts [1–4]. In sports, it can modulate arousal and emotional states, shaping both performance and experience. Nguyen and Grahn [5] found that listening to music modulates mood and arousal states, thereby influencing psychological performance. For example, after listening to Mozart, participants were able to score more points in a spatial reasoning task than participants who did not listen to music. Listening to music is also often successfully used to reduce stress [6] or by athletes in leisure gyms to improve training performance [7]. Performance benefits of music have been reported across various sports, including running [8], cycling [9], and weightlifting [10]. However, listening to music does not exclusively lead to positive outcomes; depending on the listener’s personality, context, and the music’s characteristics, it can also evoke negative emotional responses such as sadness, tension, or anxiety [11].

Although numerous studies demonstrate that music affects psychological and physical parameters, its optimal use in performance contexts remains unclear [12]. A key factor appears to be music preference, that is, whether individuals listen to self-selected or externally imposed music. Preference influences emotional valence, arousal, and attentional focus, thereby modulating psychophysiological responses and exercise performance [3].

If the music is perceived as pleasant, calming, or relaxing, better memory performance has been observed than if the music is perceived as unpleasant, exciting, or aggressive [5]. Previous research shows that music tempo and intensity affect performance, with faster music eliciting stronger physiological activation and slower music promoting relaxation [13]. Preferred music has been linked to enhanced hippocampal activity [14], improved work performance [15], and elevated mood [16]. According to Karageorghis and Priest [17], these effects are modulated by rhythmicity, familiarity, and personal preference, which collectively influence arousal and attentional focus. Preferred music enhances dopaminergic and sympathetic activity, resulting in higher motivation, reduced perceived exertion (RPE), and improved motor output [4].

In athletic contexts, music is often used to enhance motivation and mitigate perceived exertion [7, 15, 17, 18]. However, existing studies rarely distinguish between the effects of preferred and non-preferred music or examine moderating factors such as sex and age. Previous meta-analyses [3, 4, 19] focused mainly on generic music effects without accounting for individual preference. The present meta-analysis addresses this gap by comparing preferred, non-preferred, and no music conditions in psychological and physical outcomes and by examining potential moderators such as sex, age, and timing of music exposure.

Thus, this meta-analysis aimed to investigate the effects of “preferred music listening” (PML) versus “non-preferred music listening” (NPML) or “no music listening” (NML) on psychological and physical parameters in adolescent and adult recreational and competitive athletes. The parameters examined include psychological outcomes (RPE, motivation, affective response) as subjective measures and physical outcomes (strength endurance, power output, maximal strength, aerobic endurance, and speed) as objective performance indicators.

Based on previous evidence, two hypotheses were proposed: (a) PML significantly increases psychological and physical performance during the exercises performed compared to NPML, making PML more effective and preferable for sports settings, and (b) NML underperforms in all parameters when compared to both PML and NPML.

## Methods

This systematic review was prospectively registered on PROSPERO (CRD420251083551). We followed the recommendations of the PRISMA (Preferred Reporting Items for Systematic Reviews and Meta-Analysis) guidelines (Table S1) [20].

### Literature search

A systematic computerized literature search was conducted in Embase, PubMed, PsycINFO, and MEDLINE using a Boolean search strategy, following evidence-based recommendations for optimal database selection [21, 22]. For the full list of search terms, see Text S1. The literature search included studies published in English between 1 January 1960 and 12 July 2025. The broad time frame was chosen for historical completeness, as the first controlled trials on music and exercise emerged in the 1960s. However, only studies published after 1990 met the methodological standards for inclusion. Reference lists of all included studies were also screened to identify additional eligible articles. To reduce the risk of publication bias, grey literature (e.g., conference abstracts, theses) and registered but unpublished trials were also screened in trial registries and Google Scholar. However, they were excluded from the quantitative synthesis if they lacked peer-reviewed data. The term “no music” was not included as a separate search keyword, as such conditions typically represent control groups within eligible experimental studies rather than a primary search term. These studies were identified based on their comparative design, including both music and no-music conditions.

### Selection criteria

The selection criteria were based on the PICOS model (Population, Interventions, Comparators, Outcomes, Study design) [23], which is shown in Table 1. The criteria were defined as follows: (a) Population: Individuals over 14 years of age; (b) Intervention: At least one group with PML during physical activity, set-rest or warm-up; (c) Comparison: At least one group with NPML and/or NML during physical activity, set-rest, or warm-up; (d) Outcome: At least one measure of psychological or physical performance; d) Study design: Within-subjects study design (repeated-measures or crossover) with assessments in PML and NPML or NML.

**Table 1:** Overview of the applied inclusion and exclusion criteria

Category	Inclusion criteria	Exclusion criteria
Population	individuals over 14 years of age	participants had hearing impairments or severe auditory conditions (e.g., tinnitus)
Intervention	PML during physical activity, set-rest, or warm-up	inaccurate or incomplete data reporting (i.e., no measures of central tendency or variability in the results)
Comparator	NPML and/or NML during physical activity, set-rest, or warm-up	effects were examined without another comparator condition, or unspecified music preference (e.g., continuous scales)
Outcome	at least one measure of psychological or physical performance	study methodologies did not incorporate assessments of parameters for psychological or physical performance
Study design	within-subjects study design with assessments in PML and NPML or NML	between-subjects design or reviews

The criteria for excluding studies from the analysis were defined as follows: (a) participants had hearing impairments or severe auditory conditions (e.g., tinnitus); (b) data reporting was inaccurate or incomplete, specifically lacking any measures of central tendency or variability in the results; (c) effects were examined without another comparator condition, or unspecified music preference (e.g., continuous scales); (d) the study methodologies did not incorporate assessments for psychological or physical outcomes; (e) the study was of a between-subjects design or was a review article.

Only studies involving participants aged ≥ 14 years were included to avoid developmental bias in music perception and exercise physiology, as adolescence marks a phase of ongoing maturation in auditory and cognitive functions [24].

Participants were not limited to athletes, as the psychophysiological effects of music on motivation, affect, and perceived exertion are well-documented across both trained and untrained populations [4].

The intervention without music was included in a separate meta-analysis to investigate the comparison between music in general and the complete absence of auditory stimuli during physical activity, set-rest, or warm-up.

A designation other than listening to preferred or non-preferred music was only accepted as long as the method was identical, i.e., if the determination of preferred versus non-preferred music was made after subjects had communicated their choice of music in advance. The preferred choice of music was assumed to be the favorite music, motivational music, or desired music. The designation of the study group as “listening to motivational music” was therefore accepted in the studies. The control groups of the studies used with the designation “neutral music” and “podcast music”, as well as “low-motivating music”, were not accepted, as these are not comparable to the identical methodology of the music selection of the control groups from other studies used with the designation non-preferred music. Between-subject designs were excluded to minimize interindividual variability in music preference and performance responsneines.

### Assessment of methodological study quality

To evaluate the methodological quality of each study and minimize bias risk, two independent authors (PM, MN) assessed all eligible articles using the Scottish Intercollegiate Guidelines Network (SIGN) checklist for randomized controlled trials [25]. Any disagreements were resolved through consultation with a third author (JS). Inter-rater reliability for the quality assessment was calculated on a dichotomous scale using percentage agreement and Cohen’s kappa coefficient (*κ*). Agreement between the two reviewers was high (percentage agreement = 91.7%; κ = 0.764), indicating substantial reliability. Studies were classified as low quality (-), acceptable quality (+), or high quality (++). Studies deemed unacceptable (0) were excluded from analysis. The quality assessment results are provided in Table S4.

### Synthesis of results

The studies included in this meta-analysis were screened for the relevant outcome variables. To address the variability in test procedures commonly reported in the literature, both primary and alternative outcome measures were identified and are summarized in Table 2. This approach was employed to minimize the heterogeneity among the studies and ensure clearer comparability of the results.

**Table 2:** Overview of the preferred and alternative outcomes by category

Category	Preferred outcome	Alternative outcome
Rate of perceived exertion	Borg Scale (6–20) (*n* = 15)	Borg Scale (0–10) (*n* = 15)
Motivation	Visual Analog Scale (VAS 0-100; *n* = 11)	Visual Analog Scale (VAS 0–10; *n* = 1)
Affective response	Feeling Scale (FS; *n* = 4)	Physical Activity Enjoyment Scale (PACES; *n* = 3)
Strength endurance	Repetitions to failure (RTF; *n* = 12)	Total repetitions (*n* = 1) Exercise duration (*n* = 1)
Power output	Wingate test (*n* = 9)	Peak power output (*n* = 2) 1RM watt (*n* = 2)
Maximal strength	Maximal Voluntary Isometric Contraction (MVIC; *n* = 4)	One-repetition Maximum (1RM; *n* = 3)
Aerobic endurance	Total running distance (*n* = 3)	Total cycling distance (*n* = 1)
Speed	Peak sprint time (*n* = 2)	Running speed (*n* = 1) Pedal cadence (*n* = 1) Contact time (*n* = 1) Time to completion (*n* = 1)

The test data included parameters such as study days, distance covered, the number of sets in strength exercises, repetitions per set, the duration of the test performance, and intensity levels during testing. If studies did not provide detailed outcome data [26–38], a plot digitizer was used, which could extract a response range from a retrieved graph [39]. Graphical data were extracted independently by two raters (MN, PM) using WebPlotDigitizer v4.5, achieving a mean inter-rater difference of < 2.5%, ensuring accuracy and reproducibility.

To ensure consistency across heterogeneous datasets, standardized selection rules were applied when multiple measurements or testing conditions were reported within a study. De Abreu Araujo et al. [40] measured strength endurance of different muscle groups, with the leg extensors being the largest muscle group tested, they were selected for further analysis. In Latocha et al. [41], power output was measured using both the bench press and back squat; however, the back squat was chosen for analysis due to its involvement of multiple joints and muscle groups. For studies that measured multiple sets [42, 43], the best result was used for analysis. Studies with multiple independent cohorts or conditions [35, 44] were treated as separate entries in the analysis. Bentouati et al. [27] reported test results taken both in the morning and evening, but the evening data were chosen for analysis because, while no conclusive evidence exists regarding better overall performance at either time, the majority of athletes tend to engage in sports during the evening [45].

### Statistical analyses

All statistical analyses were conducted using R version 4.4.1 (R Foundation for Statistical Computing, Vienna, Austria). All outcomes were expressed as standardized mean differences (SMD) with 95% confidence intervals (CI), calculated as the difference between post-test means of the PML and comparison conditions (NPML, NML), standardized by the pooled standard deviation [46]. Positive SMD values indicate more favorable outcomes for the PML condition compared to NPML or NML. When both pre- and post-intervention data were available, post-test values were used to ensure methodological consistency across within-subject comparisons. Within-condition pre–post changes were not synthesized, and all SMDs reflect contrasts between experimental conditions at post-test.

A random-effects model was applied across all analyses, as it accounts for both within-study and between-study variability, providing a more generalized interpretation of results across diverse study conditions. Heterogeneity among studies was assessed using the *I²* statistic, with classifications based on Deeks et al. [47]: 0–40% as trivial, 30–60% as moderate, 50–90% as substantial, and 75–100% as considerable heterogeneity. Between-study variance (*τ²*) and 95% prediction intervals were also computed to provide a more comprehensive assessment of heterogeneity. All outcomes were standardized to ensure consistent directionality, where positive SMDs indicate superior performance or more favorable responses in the PML condition.

For outcomes without an overall value reported but with multiple measurements, the arithmetic mean and pooled standard deviation were calculated to derive a representative outcome for analysis [48]. Univariate random-effects meta-regressions using the Knapp–Hartung adjustment and Restricted Maximum Likelihood estimation were applied to examine the influence of study-level moderators on effect size variability. The following variables were included: participants’ age, sex, music choice (individually vs. non-individually chosen), music timing (during warm-up vs. during exercise), and condition (non-preferred vs. no music listening). Sex was operationalized as a continuous variable, calculated as the number of female participants divided by the total sample size for each study. Multivariate models were only computed when at least ten studies per moderator were available [49].

Sensitivity analyses using a leave-one-out procedure showed that excluding individual studies did not meaningfully alter the overall effect size, indicating that no single study disproportionately influenced the pooled results (Additional File 2, Figures S1–S8). To assess publication bias, funnel plots for each outcome were visually inspected, and Egger’s regression test was applied to statistically examine asymmetry (*p* < 0.05 indicating potential bias). In addition, the trim-and-fill method [50] was used to estimate the potential impact of unpublished studies on pooled effect sizes.

## Results

**Fig. 1:**
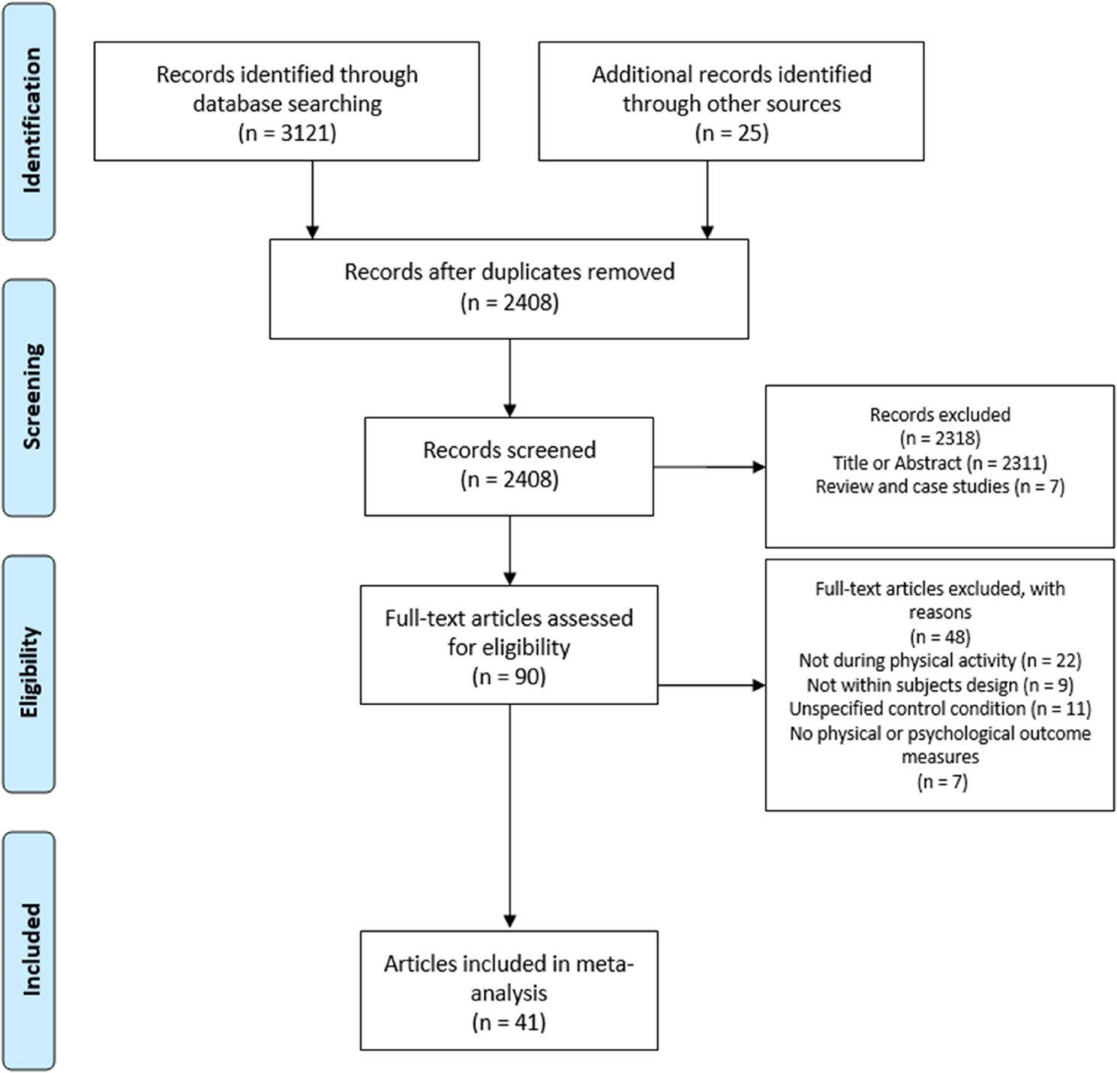
Flow diagram of the systematic literature search

Figure 1 summarizes the process of the systematic literature search. A total of 3121 records were identified through database searching, and an additional 25 through other sources. After removal of duplicates, 2408 records were screened. Of these, 2318 records were excluded based on title and abstract screening, leaving 90 full-text articles for eligibility assessment. The final screening of these 90 records was independently conducted by two reviewers (MN, JS), with very high inter-rater agreement (95.3%; Cohen’s *κ* = 0.825), indicating substantial reliability. Discrepancies were resolved by consensus with a third reviewer (RB). A total of 48 full-texts were excluded for predefined reasons. Ultimately, 41 studies met all inclusion criteria and were included in the meta-analysis.

### Study characteristics

The characteristics of the included studies are summarized in Table S2, detailing the authors, year of publication, participant demographics, intervention and control groups, study design, the types of interventions applied, test and music modality, outcome measures, main findings for each group, and methodological quality. Of the 41 included studies, all investigated the effects of PML, 14 studies [26, 27, 31, 33, 36, 38, 43, 51–57] examined the effects of NPML​, and 36 studies [8, 27–32, 34–38, 40–44, 51, 54, 56–70] analyzed the impact of NML​. In terms of study design, 17 studies [27, 29, 31, 33, 35–37, 40, 44, 52, 53, 55, 56, 58, 60, 63] employed a randomized crossover design, 14 [8, 30, 32, 41, 42, 51, 54, 61, 62, 64, 65, 68, 70, 71] a randomized repeated-measures design, five [28, 34, 43, 59, 66] a non-randomized repeated-measures design, and four [38, 57, 67, 69] a non-randomized crossover design.

### Participant characteristics

Collectively, the 41 studies included a total of 855 participants, comprising 282 females and 573 males. Regarding psychological performance, 553 participants (130 F, 423 M) were tested for RPE, 192 (71 F, 121 M) for motivation, and 196 (50 F, 146 M) for affective response. In terms of physical performance, 241 participants (42 F, 199 M) performed strength endurance tasks, 181 (54 F, 127 M) completed power output tests, 353 (127 F, 226 M) were assessed for maximal strength, 235 (56 F, 179 M) for aerobic endurance, and 145 (6 F, 139 M) for speed. Participants were predominantly healthy adults with mixed training backgrounds, ranging from insufficiently active or recreationally active individuals to trained athletes, reflecting the heterogeneity of study populations. The participants engaged in various physical activities such as resistance training, running, cycling, CrossFit, basketball, soccer, and volleyball. Ages ranged from 14 to 65 years​​.

### Music modality characteristics

In the PML condition, participants typically selected their own preferred music from favorite genres or personal playlists. The tempo of the chosen music generally ranged between 95 and 180 bpm, with most studies reporting values above 120 bpm. Lower tempo selections were noted in Greco et al. [28] with 107.4 ± 42.4 bpm, Hutchinson et al. [55] with 119 ± 11 bpm, Tanaka et al. [69] with < 120 bpm, and Blasco–Lafarga et al. [61] with 116.0 ± 17.3 bpm. Six studies [38, 41, 51, 60, 62, 70] did not report bpm values.

Volume levels were either self-selected or standardized, though procedures varied between studies. Eleven studies [30, 37, 42, 44, 54, 55, 57, 60, 61, 69] reported volume values, which generally ranged between 70 and 90 dB. The remaining 31 studies [8, 26–28, 31–36, 38, 40, 41, 43, 51–53, 56, 58, 59, 62–68, 70, 71] did not report specific volume data.

In the NPML condition, music was selected by researchers and comprised genres or tracks previously identified as unpreferred by participants (see Additional File 1 for study-level details). Selection procedures, however, varied across studies. Tempo was usually matched to the PML condition to isolate the effect of music preference. However, Nakamura et al. [43] deviated from this approach, using a slower tempo in the NPML condition (95 ± 28 bpm) compared to the PML condition (117 ± 29 bpm).

The NML condition served as a passive control in which participants performed the tasks without any musical input. Five studies [26, 33, 52, 53, 55] did not include an NML condition, analyzing only the effects of PML and NPML.

### Outcome characteristics

The outcome measures in this meta-analysis were categorized into psychological and physical parameters. The psychological parameters included RPE, which was assessed in 30 studies [8, 26–28, 30–35, 42–44, 51, 52, 55–57, 60–65, 67–70], motivation, measured in 11 studies [26, 31, 33, 35–37, 52, 53, 55, 58], and affective response, evaluated in nine studies [28, 30, 32, 34, 37, 42, 51, 62, 69].

RPE was consistently measured using the Borg Scale, either the 6–20 or 0–10 version. This scale has been widely validated for use in exercise performance contexts [72, 73]. Motivation was assessed using a visual analog scale (VAS), ranging from 0 to 100 mm or 0 to 10 mm. The VAS has been demonstrated to be a reliable tool for measuring subjective motivation in physical tasks [74, 75]. Affective response was measured, primarily using the Physical Activity Enjoyment Scale (PACES) and the Feeling Scale (FS), both of which are validated for assessing emotional states in exercise settings [76, 77].

Regarding physical parameters, strength endurance was examined in 13 studies [26, 30, 40, 51, 53, 55–60, 62, 66], power output in 13 studies [27, 31–33, 35–37, 41, 52, 53, 58, 70], maximal strength in six studies [28, 36, 38, 56, 57, 59, 66], speed in six studies [29, 30, 63, 65, 68], and aerobic endurance in four studies [43, 54, 64, 65].

Strength endurance, defined as the number of repetitions performed to failure, was primarily measured using the barbell bench press and has been validated as a measure of muscular endurance in resistance training [78]. Power output was measured primarily using Wingate tests, which is recognized for reliability and validity in measuring peak power output during dynamic activities [79]. Maximal strength was assessed using MVIC and 1RM tests, which both have demonstrated strong reliability in multiple contexts [80, 81], however presenting different estimations of maximal strength capacity [82]. Additionally, aerobic endurance was recorded using standard distance protocols in running and cycling, and speed was measured, with both variables commonly assessed using timing systems such as electronic timing gates or global positioning systems, which are validated for measuring exercise performance [83].

### Methodological study quality

**Fig. 2:**
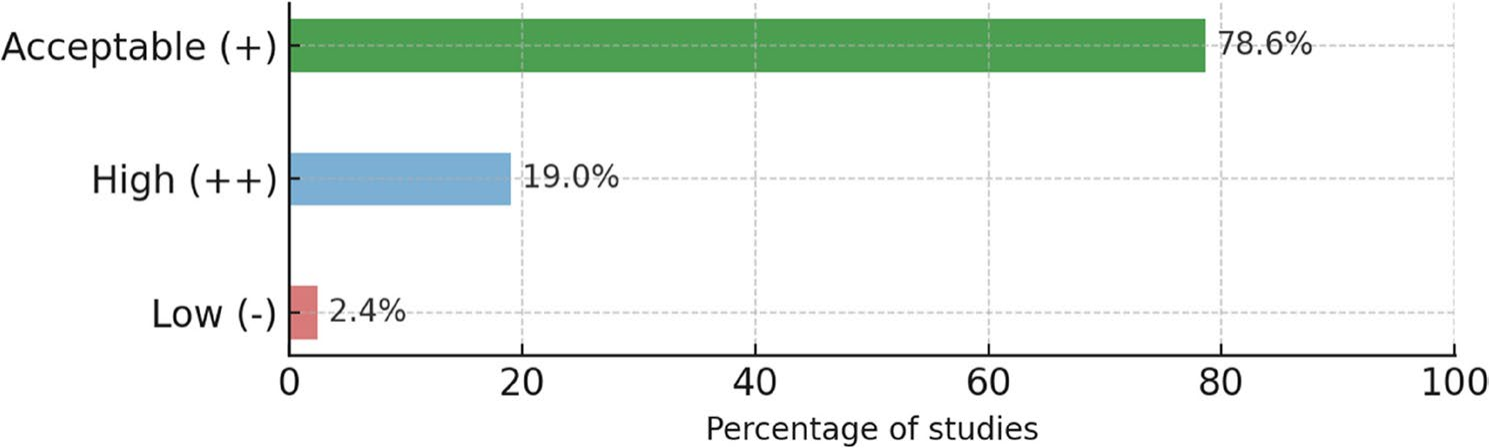
Distribution of methodological study quality according to the Scottish Intercollegiate Guidelines Network (SIGN) checklist

Most studies were rated as acceptable quality (+, 78.6%), followed by high quality (++, 19.0%) and low quality (−, 2.4%) according to the SIGN checklist (Table S3). Figure 2 illustrates the distribution of methodological study quality across all included studies. Overall, the methodological rigor of the evidence base was high, with no study rated as unacceptable (0).

### The effects of music on psychological parameters

**Fig. 3:**
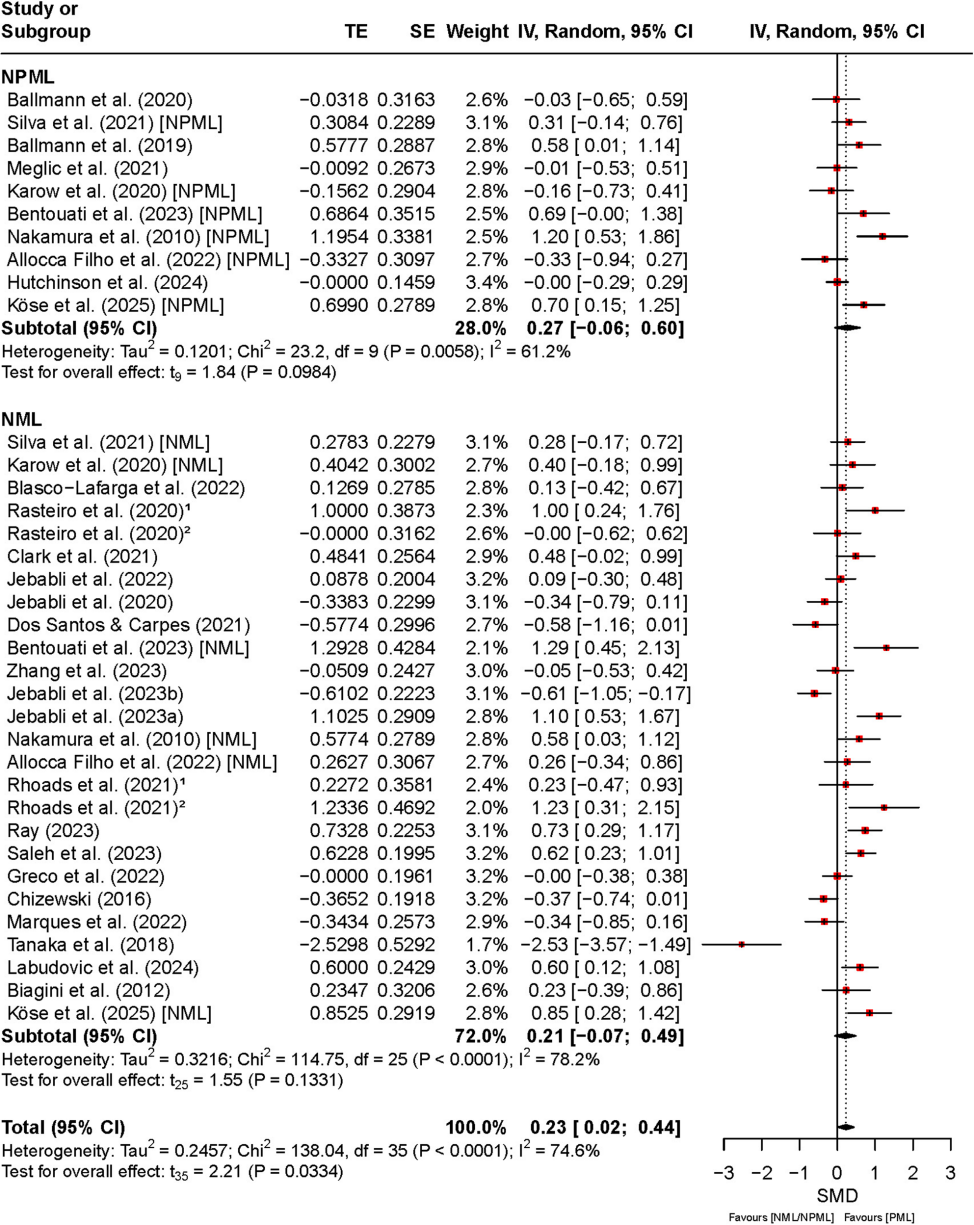
Comparison of the effect between preferred and non-preferred/no music listening on RPE. Note: CI: confidence interval; df: degrees of freedom; IV: inverse variance; NML: no music listening; NPML: non-preferred music listening; PML: preferred music listening; TE: treatment effect; SE: standard error

Figure 3 illustrates the effects of PML, NPML, and NML on RPE. The pooled analysis indicated a statistically significant reduction in perceived exertion under PML compared to NPML and NML (SMD = 0.23, 95% CI [0.02, 0.44], *t*₃₅ = 2.21, *p* = 0.03). The CIs suggest that the effect was consistent across studies. Heterogeneity was moderate to high (*I*² = 75%, *p* < 0.01). In the NPML subgroup, the effect was similar in magnitude (SMD = 0.27, 95% CI [–0.06, 0.60]), but the CI included zero, indicating greater uncertainty and a less robust effect estimate. Variability among these studies was moderate (*I*² = 61%), reflecting methodological diversity and smaller sample sizes.

**Fig. 4:**
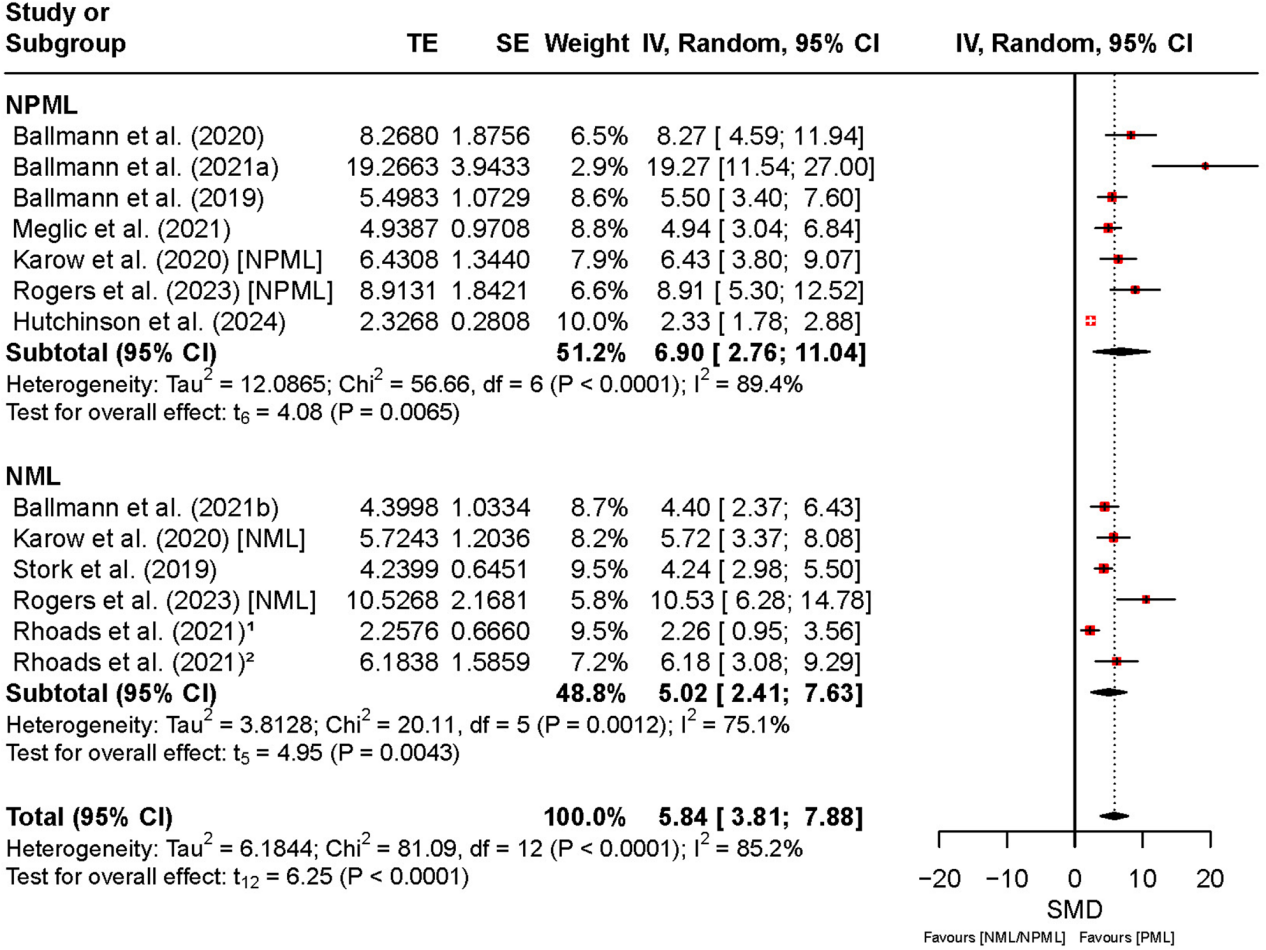
Comparison of the effect between preferred and non-preferred/no music listening on motivation. Note: CI: confidence interval; df: degrees of freedom; IV: inverse variance; NML: no music listening; NPML: non-preferred music listening; PML: preferred music listening; TE: treatment effect; SE: standard error

Figure 4 summarizes the effects of PML, NPML, and NML on motivation. The overall pooled effect was statistically significant (SMD = 5.84, 95% CI [3.81, 7.88], *t*₁₂ = 6.25, *p* < 0.01), indicating a significant improvement in motivation under PML compared to both NPML and NML. The CI suggests a consistently positive effect, though with notable variability across studies (*I*² = 85%).

In the subgroup analysis, NPML showed a comparable effect (SMD = 6.90, 95% CI [2.76, 11.04], *p* < 0.01), but the wide CI indicates greater uncertainty in the magnitude of this estimate. For NML, the effect was statistically significant (*SMD* = 5.02, 95% CI [2.41, 7.63], *p* < 0.01), with slightly lower heterogeneity (*I*² = 75%). Overall, while PML demonstrated a positive effect on motivation, the high heterogeneity (*I*² = 75–89%) reflects considerable variability between studies, likely due to methodological and participant differences.

**Fig. 5:**
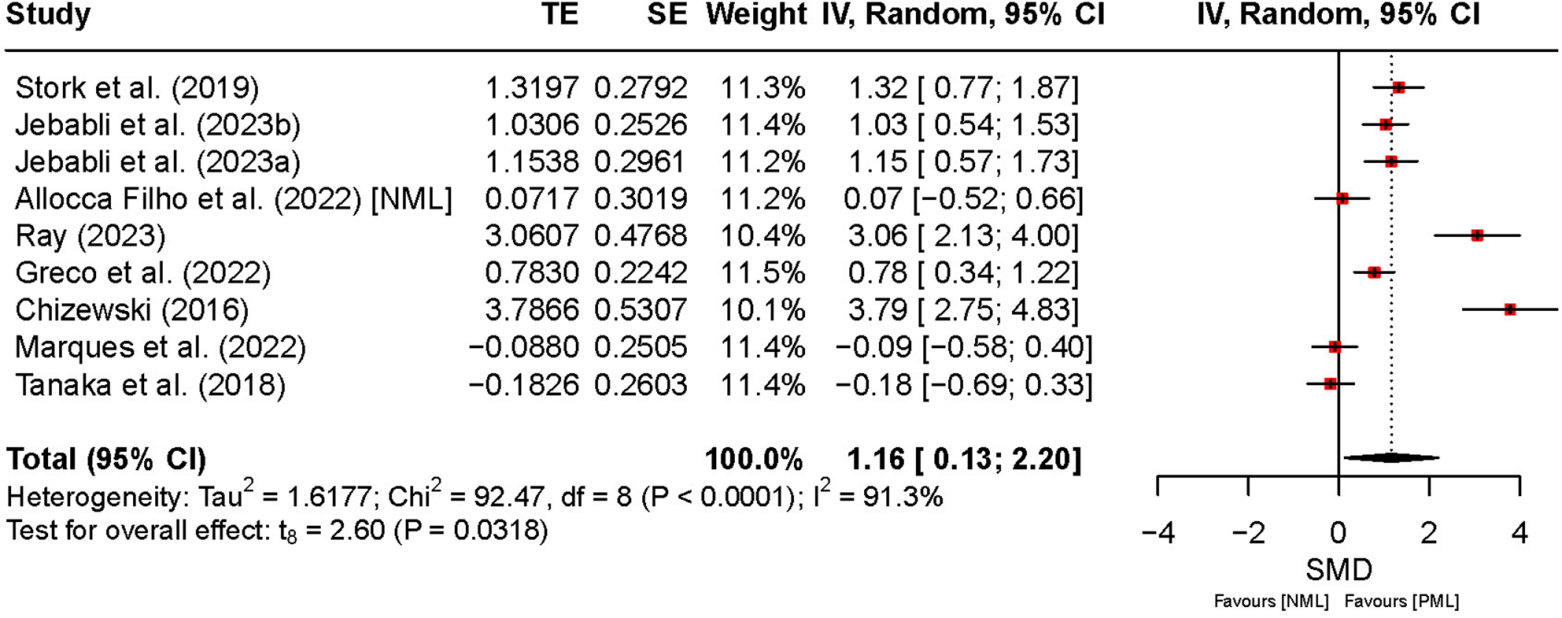
Comparison of the effect between preferred and non-preferred/no music listening on affective response. Note: CI: confidence interval; df: degrees of freedom; IV: inverse variance; NML: no music listening; NPML: non-preferred music listening; PML: preferred music listening; TE: treatment effect; SE: standard error

Figure 5 presents the effects of PML, and NML on affective response. The overall pooled effect indicated a significant positive effect in affective response under PML compared to NML (SMD = 1.16, 95% CI [0.13, 2.20], *t₉* = 2.60, *p* = 0.03), while the relatively wide CI and high heterogeneity (*I*² = 91%) suggest variability in effect magnitude across studies. As only one study [51] reported NPML data for affective response, no pooled effect was calculated for this subgroup. Heterogeneity was substantial across studies (*I*² = 92%, *p* < 0.01), reflecting marked between-study differences. While PML generally improved affective response, the large heterogeneity implies that individual preferences and methodological variations considerably influenced these outcomes.

### The effects of music on physical parameters

**Fig. 6:**
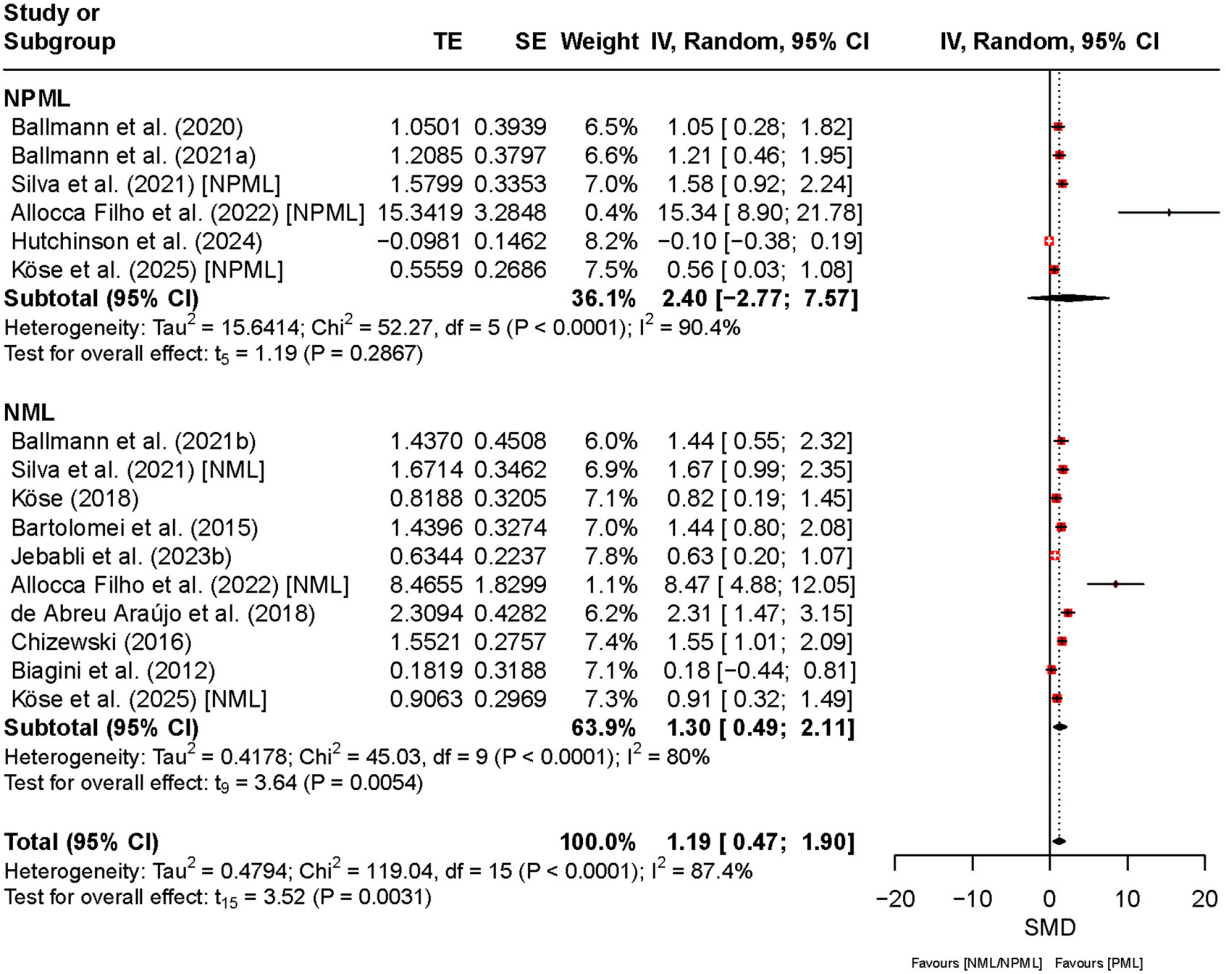
Comparison of the effect between preferred and non-preferred/no music listening on strength endurance. Note: CI: confidence interval; df: degrees of freedom; IV: inverse variance; NML: no music listening; NPML: non-preferred music listening; PML: preferred music listening; TE: treatment effect; SE: standard error

Figure 6 presents the effects of PML, NPML, and NML on strength endurance. The overall SMD was 1.19 (95% CI [0.47, 1.90], *t*₁₅ = 3.52, *p* < 0.01), indicating a significant improvement in strength endurance under PML compared to NPML and NML. The CI, though relatively wide, consistently favors PML and supports a positive effect. Heterogeneity was high (*I*² = 87%, *p* < 0.01), suggesting substantial variability between studies. For NPML, the SMD was 2.40 (95% CI [–2.77, 7.57]), but the wide CI spanning zero indicates large uncertainty in the pooled effect, likely reflecting inconsistent study outcomes. Heterogeneity was high (*I*² = 90%), reflecting expected between-study variability. For NML, the SMD was 1.30 (95% CI [0.49, 2.11]), with similarly high heterogeneity (*I*² = 80%), indicating that despite a positive trend, variability between studies remains a key limitation in interpreting these results.

**Fig. 7:**
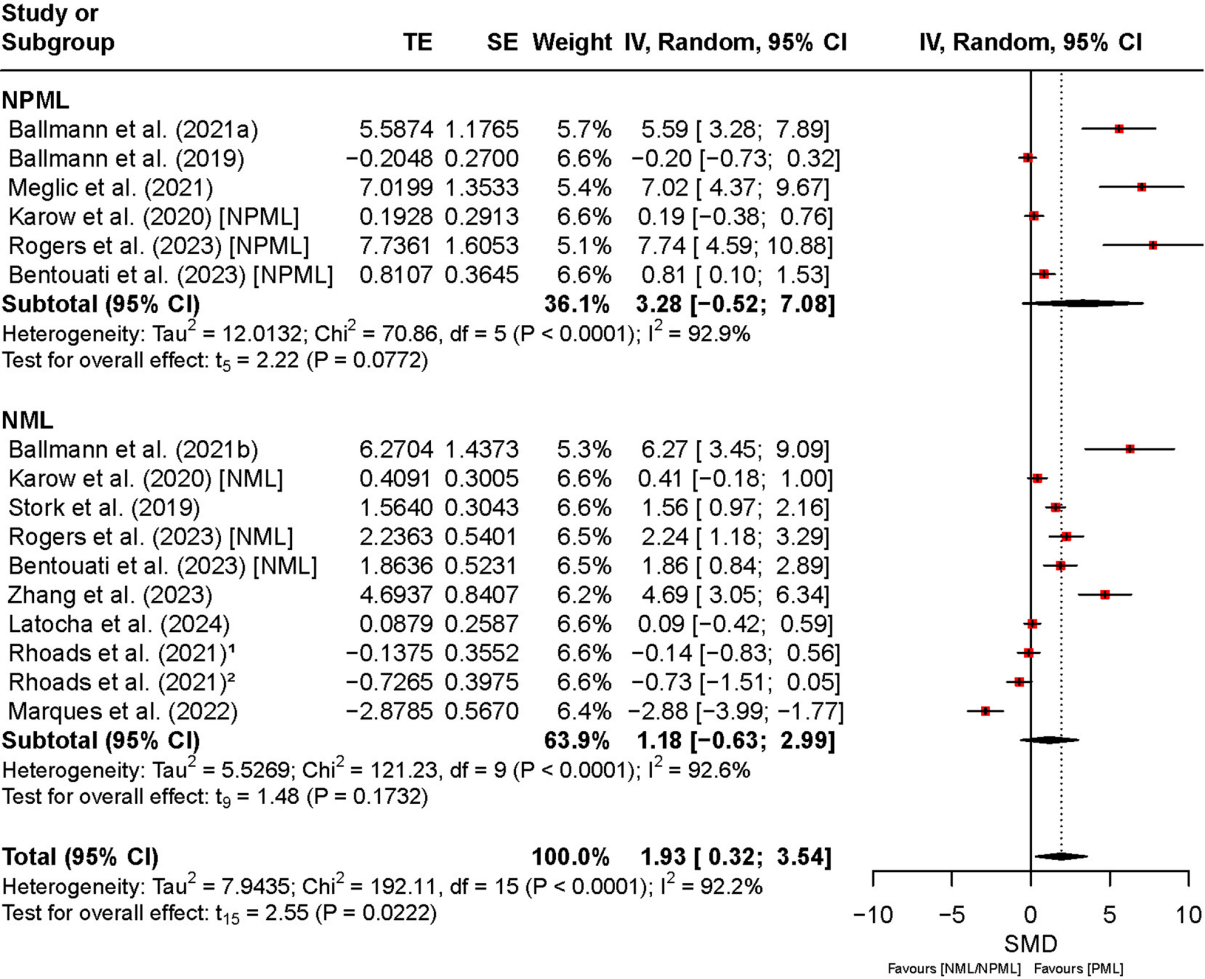
Comparison of the effect between preferred and non-preferred/no music listening on power output. Note. CI: confidence interval; df: degrees of freedom; IV: inverse variance; NML: no music listening; NPML: non-preferred music listening; PML: preferred music listening; TE: treatment effect; SE: standard error

Figure 7 shows the effects of PML, NPML, and NML on power output across the included studies. The overall SMD was 1.93 (95% CI [0.32, 3.54], *t*₁₅ = 2.55, *p* = 0.02), indicating a significant improvement in power output under PML compared to NPML and NML. Although the effect was statistically significant, the wide CI reflects variability and uncertainty across studies. Heterogeneity was very high (*I*² = 93%, *p* < 0.01), indicating between-study differences. For NPML, the SMD was 3.28 (95% CI [–0.52, 7.08]), and the inclusion of a negative lower bound suggests inconsistent and potentially unstable effects across studies. Similarly, for NML, the SMD was 1.18 (95% CI [–0.63, 2.99]), indicating that the effect was not consistently positive. Both subgroup comparisons showed high heterogeneity (*I*² = 93%), reflecting methodological or population-related variability that limits the generalizability of these findings.

**Fig. 8:**
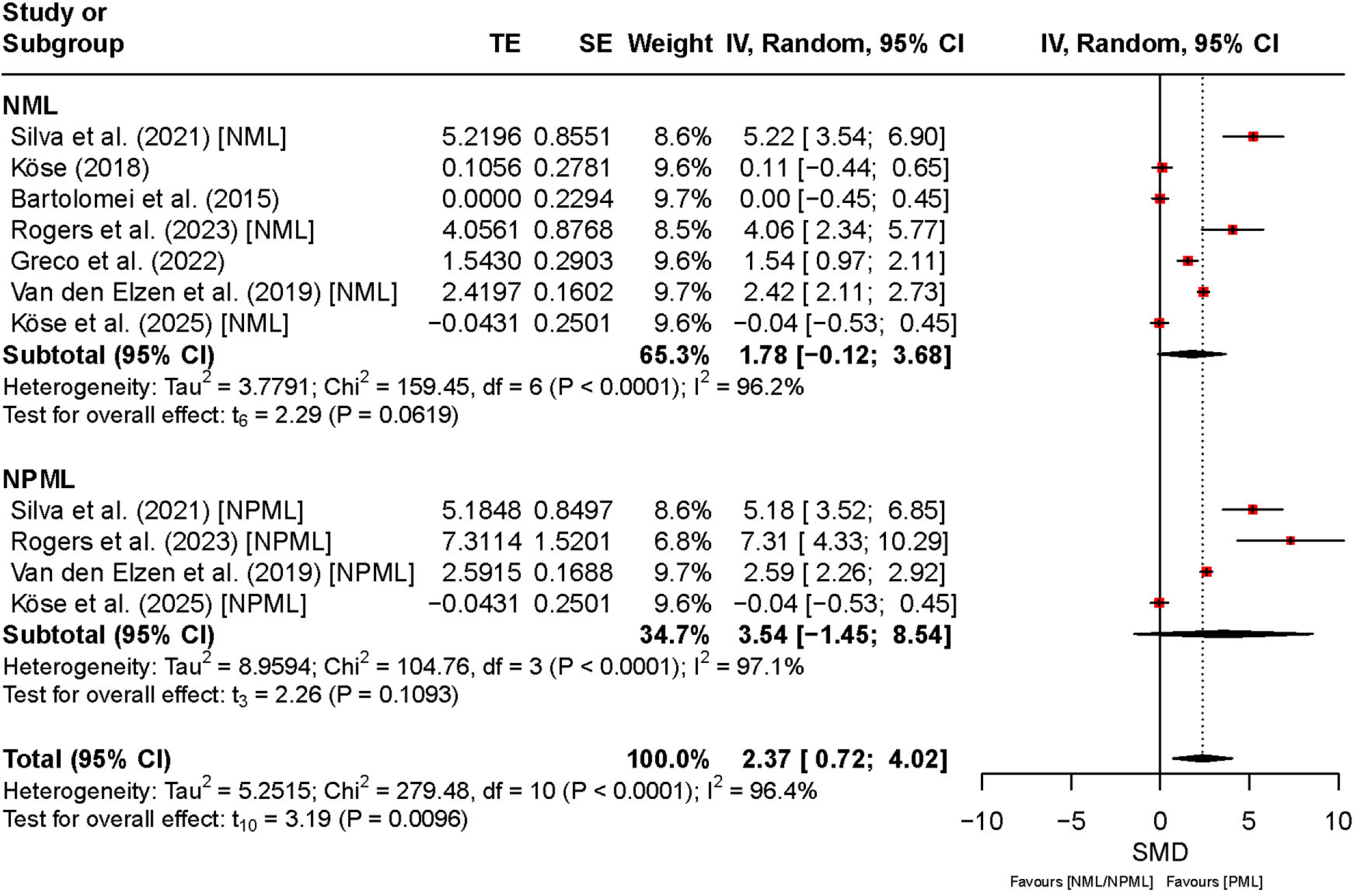
Comparison of the effect between preferred and non-preferred/no music listening on maximal strength. Note: CI: confidence interval; df: degrees of freedom; IV: inverse variance; NML: no music listening; NPML: non-preferred music listening; PML: preferred music listening; TE: treatment effect; SE: standard error

Figure 8 shows the effects of PML, NPML, and NML on maximal strength. The overall SMD was 2.37 (95% CI [0.72, 4.02], *t*₁₀ = 3.19, *p* < 0.01), indicating a significant improvement in maximal strength under PML compared with NPML and NML. However, the wide CI and very high heterogeneity (*I*² = 96%, *p* < 0.01) indicate that the magnitude of this effect varied substantially across studies. For NPML, the SMD was 3.54 (95% CI [–1.45, 8.54]), and the broad interval, including a negative lower bound, suggests marked inconsistencies and limited precision of the pooled estimate. Similarly, for NML, the SMD was 1.78 (95% CI [–0.12, 3.68]), implying that the direction and magnitude of the effect were not consistent across studies. Both subgroup analyses showed high heterogeneity (*I*² = 97% and *I*² = 96%), reflecting expected between-study variability and methodological diversity.

**Fig. 9:**
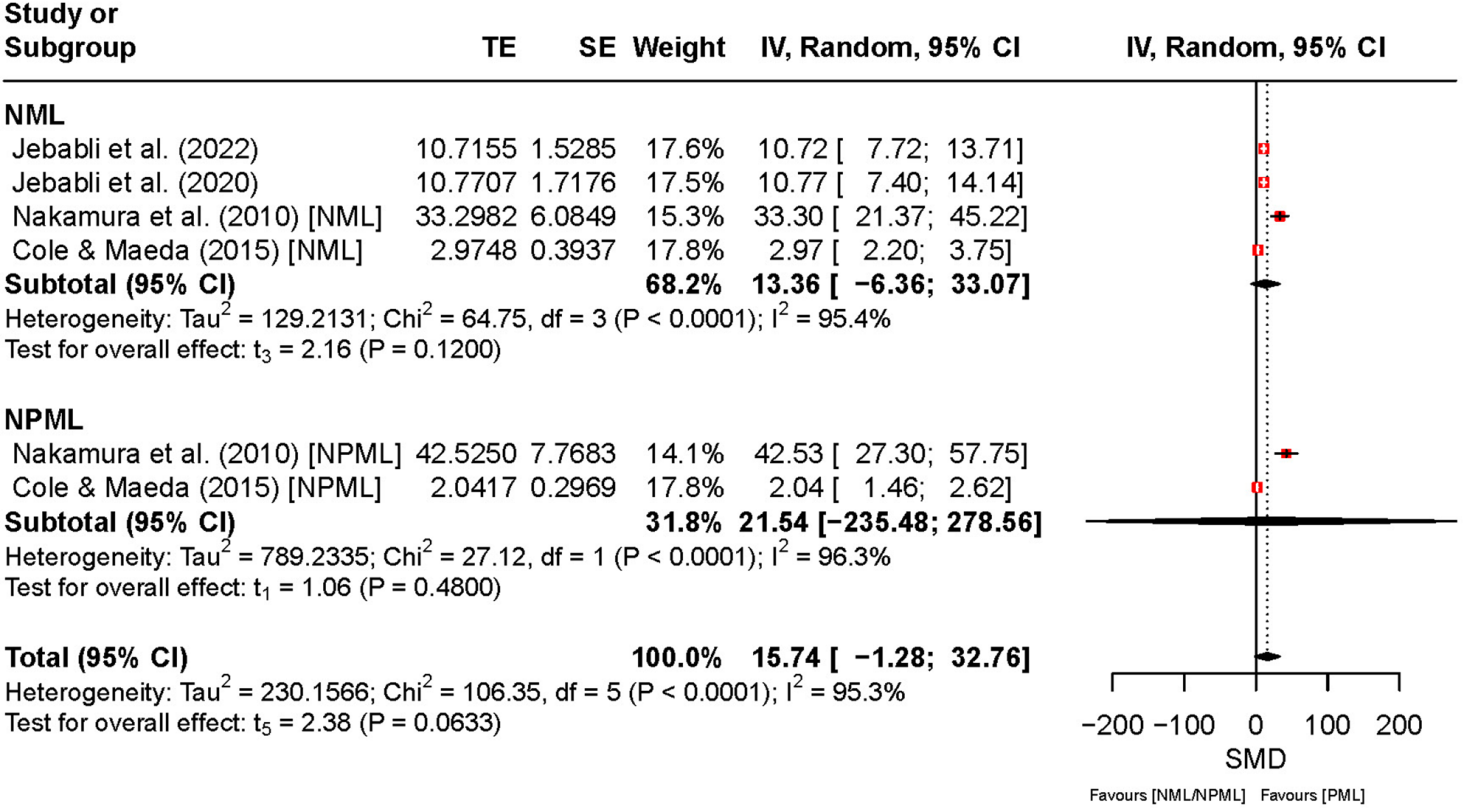
Comparison of the effect between preferred and non-preferred/no music listening on aerobic endurance. Note. CI: confidence interval; df: degrees of freedom; IV: inverse variance; NML: no music listening; NPML: non-preferred music listening; PML: preferred music listening; TE: treatment effect; SE: standard error

Figure 9 presents the effects of PML, NPML, and NML on aerobic endurance. The overall SMD was 15.74 (95% CI [–1.28, 32.76], *t*₅ = 2.38, *p* = 0.06), suggesting a trend toward improved aerobic endurance under PML compared to NPML and NML, although this effect did not reach statistical significance. The wide CI and very high heterogeneity (*I*² = 95%, *p* < 0.01) indicate substantial between-study variability and uncertainty in the pooled estimate. For NPML, the SMD was 21.54 (95% CI [–235.48, 278.56], *I*² = 96%), reflecting extremely inconsistent results across studies, with confidence bounds spanning both large positive and negative effects. Similarly, for NML, the SMD was 13.36 (95% CI [–6.36, 33.07], *I*² = 95%), reflecting between-study variability.

**Fig. 10:**
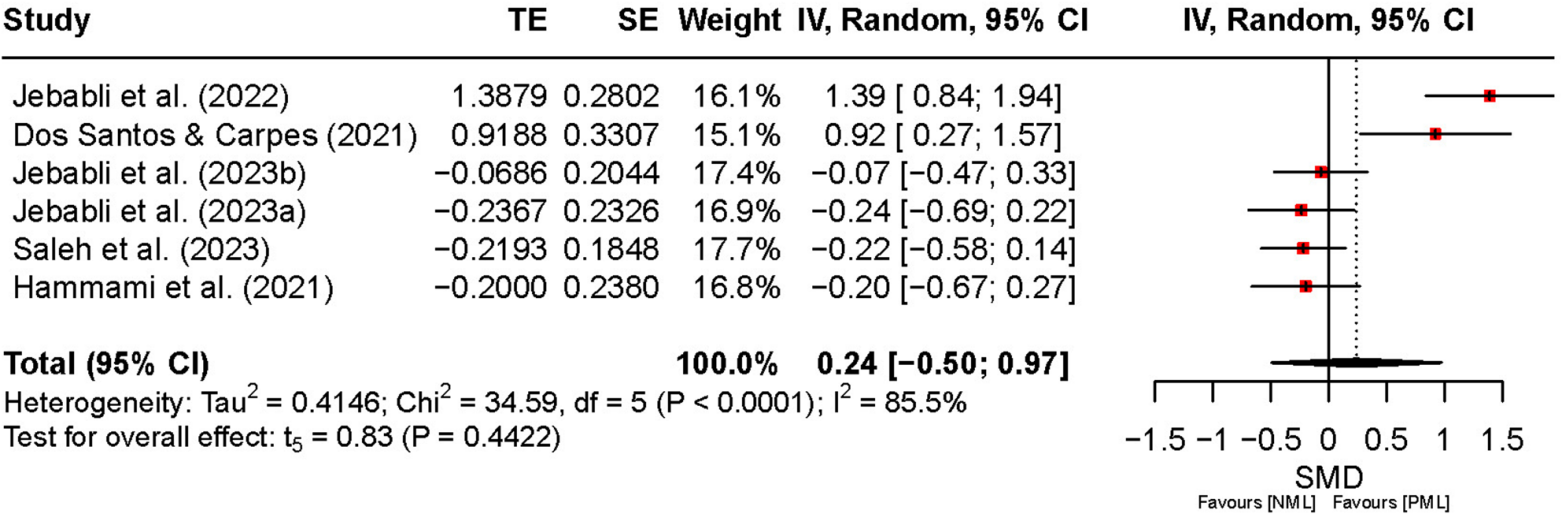
Comparison of the effect of preferred and no music listening on speed. Note: CI: confidence interval; df: degrees of freedom; IV: inverse variance; NML: no music listening; PML: preferred music listening; TE: treatment effect; SE: standard error

Figure 10 illustrates the effects of PML compared with NML on speed performance. The overall SMD was 0.24 (95% CI [–0.50, 0.97], *t*₅ = 0.83, *p* = 0.44), indicating no significant difference between conditions. The CI includes zero and spans both negative and positive effects, suggesting that the direction and magnitude of the effect vary across studies. Heterogeneity was high (*I*² = 86%, *p* < 0.01), reflecting considerable between-study variability. While some studies reported positive effects of PML on speed, the pooled estimate indicates no consistent performance benefit.

### Comparison of PML vs. NPML and NML

This analysis aimed to compare the effects of PML with NPML and NML across various psychological and physical parameters, examining the comparative effects of music preference conditions on performance outcomes.

For the psychological outcomes, motivation displayed a significant increase in the PML condition compared to both NPML and NML. The SMD for motivation was 5.84 (95% CI: [3.81, 7.88]; *p* < 0.01), indicating a substantial enhancement in motivational levels with PML. Subgroup analysis indicated that while both NPML and NML showed improvements in motivation, PML yielded the highest mean motivation scores, although subgroup differences were not statistically significant (*Q* = 0.90, *p* = 0.34).

In terms of RPE, the overall effect showed a significant difference between PML and the comparison conditions. The SMD for RPE was 0.23 (95% CI: [0.02, 0.44]; *p* = 0.03), suggesting that perceived exertion was slightly lower with PML. The variability in perceived exertion outcomes was moderate to high (*I²* = 75%). Subgroup analysis revealed no significant difference between NPML and NML (*Q* = 0.08, *p* = 0.77).

For affective response, a significant effect was found when comparing PML to NML (SMD = 1.16, 95% CI: [0.13, 2.20]; *p* = 0.03). No subgroup analysis was performed for NPML because only one study was available for this condition.

Regarding physical performance, strength endurance demonstrated a significant increase under PML. The SMD was 1.19 (95% CI: [0.47, 1.90]; *p* < 0.01), indicating that participants exhibited greater strength endurance when listening to preferred music. The effect was statistically significant in the comparison with NML (SMD = 1.30, 95% CI: [0.49, 2.11]), whereas the comparison with NPML yielded a wide and non-significant CI (SMD = 2.40, 95% CI: [–2.77, 7.57]), suggesting that the most consistent benefits of PML were observed relative to no music. Subgroup analysis revealed no significant difference between NPML and NML (*Q* = 0.29, *p* = 0.59).

Power output and maximal strength also showed significant increases with PML. For power output, the SMD was 1.93 (95% CI: [0.32, 3.54]; *p* = 0.02), while for maximal strength, it was 2.37 (95% CI: [0.72, 4.02]; *p* < 0.01). These results indicate that participants achieved higher power output and strength levels with preferred music. Subgroup analysis revealed no significant difference between NPML and NML (Power: *Q* = 1.56, *p* = 0.21; Maximal strength: *Q* = 1.02, *p* = 0.31).

For maximal strength, the effect was statistically significant in comparison to NML, while the comparison with NPML yielded a wide and non-significant CI, indicating substantial between-study variability. Subgroup analysis revealed no significant difference between NPML and NML (*Q* = 1.02, *p* = 0.31).

For aerobic endurance, PML yielded an SMD of 15.74 (95% CI: [-1.28, 32.76]; *p* = 0.06), indicating a trend toward longer distances covered compared to NPML and NML, though this result was marginally non-significant. Subgroup analysis revealed no significant difference between NPML and NML (*Q* = 0.15, *p* = 0.70).

Similarly, speed performance showed no significant difference between PML and NML (SMD = 0.24; *p* = 0.44), suggesting that while PML may aid endurance, its effect on speed alone is limited. No subgroup analysis was performed for speed, as data were available for NML only.

Overall, these findings suggest that PML has the most substantial effects on motivation, strength endurance, power output, and maximal strength. The benefits of PML are particularly prominent compared to NML, with inconsistent or less robust advantages over NPML, underscoring the potential importance of personal music preference in sports performance contexts.

### Publication bias

**Fig. 11:**
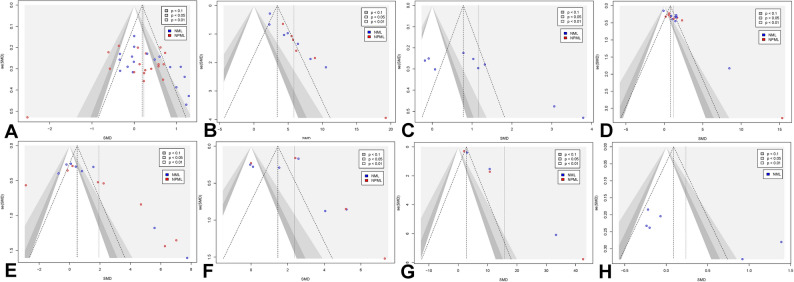
Funnel plots for publication bias assessment regarding (**A**) RPE, (**B**) motivation, (**C**) affective response, (**D**) strength endurance, (**E**) power output, (**F**) maximal strength, (**G**) aerobic endurance, and (**H**) speed. Note: SMD: standardized mean difference; SE: standard error; NML: no music listening; NPML: non-preferred music listening

To assess potential reporting bias, Egger’s regression was conducted across the analyzed outcomes, with results presented in Fig. 11A–H. Publication bias was evaluated by testing for asymmetry in the funnel plots, where a significant intercept suggests potential bias.

Egger’s regression analysis indicated significant publication bias for motivation (*t* = 10.26, *p* < 0.0001), strength endurance (*t* = 6.85, *p* < 0.0001), aerobic endurance (*t* = 15.62, *p* < 0.0001), and power output (*t* = 3.29, *p* = 0.0054). For other outcomes, no significant bias was detected: RPE (*t* = 1.17, *p* = 0.2516), maximal strength (*t* = 0.56, *p* = 0.5884), and affective response (*t* = 1.80, *p* = 0.1092) showed symmetrical distributions, indicating minimal risk of reporting bias. While speed showed minor asymmetry (*t* = 2.45, *p* = 0.0708), it was not statistically significant. Trim-and-fill analyses indicated no substantial changes in the direction or magnitude of the pooled effects, suggesting that publication bias did not meaningfully influence the overall results.

These findings suggest that small-study effects or selective reporting may have inflated the observed effects for motivation, strength endurance, aerobic endurance, and power output. As a result, the magnitude of these outcomes should be interpreted with caution. However, for the remaining outcomes, the absence of significant bias supports the robustness of the corresponding pooled estimates.

### Sensitivity analyses

To assess the robustness of the meta-analytic results, leave-one-out (L1O) sensitivity analyses were performed for all outcomes (Figures S1–S8). Overall, the omission of individual studies did not substantially affect the direction or significance of the pooled estimates.

For psychological outcomes, effect sizes for RPE (SMD = 0.23, 95% CI [0.02, 0.44], *p* = 0.03, *I²* = 75%), motivation (SMD = 5.84, 95% CI [3.81, 7.88], *p* < 0.01, *I²* = 85%), and affective response (SMD = 1.16, 95% CI [0.13, 2.20], *p* = 0.03, *I*² = 91%) remained stable across all L1O analyses.

For physical outcomes, strength endurance (SMD = 1.19, 95% CI [0.47, 1.90], *p* < 0.01, *I²* = 87%), maximal strength (SMD = 2.37, 95% CI [0.72, 4.02], *p* < 0.01, *I²* = 96%), power output (SMD = 1.93, 95% CI [0.32, 3.54], *p* = 0.02, *I²* = 92%), and speed (SMD = 0.24, 95% CI [− 0.50, 0.97], *p* = 0.44, *I²* = 86%) demonstrated similar robustness.

Only aerobic endurance (SMD = 15.74, 95% CI [− 1.28, 32.76], *p* = 0.06, *I²* = 95%) showed greater variability across study omissions, suggesting a more sensitive outcome.

Collectively, the L1O analyses support the stability of the meta-analytic findings, except for aerobic endurance, where caution in interpretation is warranted.

### Moderator variables and meta‑regression

Given the significant heterogeneity across several outcome measures (*τ²* = 0.25–789.23; *I²* = 61–96%), meta-regression analyses were performed to examine the impact of study characteristics on effect sizes in preferred music listening studies.

Across outcomes, the meta-regression models explained small to large proportions of the between-study variance (*R²* = 0–84.6%, Table S5). For perceived exertion, motivation, and most physical outcomes (maximal strength, power output, aerobic endurance, speed), none of the tested moderators reached statistical significance (all *p* > 0.05).

However, sex significantly moderated the effects for both motivation (*β* = 1.21, *p* = 0.002, *R²* = 0%) and affective response (*β* = 3.18, *p* = 0.018, *R²* = 59.8%), indicating stronger positive effects in studies including a higher proportion of female participants. In addition, a significant moderating effect of *sex* was found for strength endurance (*β* = 1.22, *p* = 0.011, *R²* = 0%), with greater benefits in male-dominant samples. No other significant moderator effects were observed.No other significant moderator effects were observed.

These findings suggest that the influence of study-level characteristics on the ergogenic and psychological effects of preferred music is generally limited. Nevertheless, the observed moderating role of sex for motivation and affective responses indicates that psychological effects of preferred music may vary depending on the sex composition of study samples, whereas physical performance measures appear largely stable across populations.

## Discussion

The effects of preferred, non-preferred, and no music listening on psychological and physical outcomes in exercise and sports were investigated in this meta-analysis. Across 41 studies including 855 participants, preferred music showed the most consistent benefits across psychological and performance-related parameters.

These findings align with established psychophysiological frameworks, suggesting that preferred music regulates arousal, attention, and affect, thereby enhancing motivation and motor output [4, 17].

Preferred music significantly enhanced motivation and moderately reduced perceived exertion, while affective responses improved mainly compared with no music. Meta-regression analyses revealed that sex moderated both motivational and affective outcomes, with stronger effects in female-dominant samples, and also influenced strength endurance, with greater benefits in male-dominant studies. No significant moderating effects of age, music choice, or music timing were found. Overall, psychological responses to preferred music appear more sensitive to interindividual characteristics, whereas ergogenic effects remain largely consistent across populations.

### Effects of music listening on physical parameters

This meta-analysis provides evidence that strength endurance, power output, and maximal strength were higher under PML compared to NPML and NML, while showing more limited or inconsistent effects on aerobic endurance and speed.

Strength endurance was significantly higher under PML, as measured by repetitions to failure, compared to both NPML and NML. This finding is consistent with the notion that music, particularly when preferred, serves as a dissociative stimulus, shifting attention away from fatigue and discomfort during repetitive, submaximal tasks [84]. By regulating arousal and mood, PML helps sustain effort for longer periods, likely explaining the higher number of repetitions to failure observed. Additionally, rhythmic elements in music may assist in movement pacing, contributing to prolonged muscular engagement [85].

Power output was significantly greater under PML compared to NPML and NML. This effect may be attributed to music’s ability to regulate psychophysiological arousal, where music modulates the athlete’s psychophysiological state toward an optimal activation zone, potentially enhancing motor unit recruitment and muscular explosiveness [86, 87]. Moreover, rhythmic cues can aid in timing and force application, which are central to power generation in dynamic movements [88].

Maximal strength was also significantly higher under PML compared to NPML and NML, as shown in tasks such as 1RM and MVIC. While maximal strength is typically less susceptible to external influences, these results further suggest that preferred music may enhance cortical excitability, motor readiness, and psychological preparedness, thus improving performance [86]. These effects are consistent with broader findings that music enhances performance in high-stakes, precision-based physical tasks. For example, El Boghdady and Ewalds-Kvist [89] found that surgeons performed better on spatial-temporal and physical tasks when listening to music than without it, suggesting that music can facilitate cognitive-motor integration even outside of sports.

While a tendency toward higher aerobic endurance was observed under PML compared to NPML and NML, the effect did not reach statistical significance. This may be due to high variability among protocols and participant fitness levels. Endurance performance is multifactorial, influenced not only by motivation and arousal but also by pacing strategy, energy availability, or training status [90]. Although music may help regulate tempo and perceived effort in steady-state conditions, its benefit may diminish over longer durations or more physiologically taxing efforts [91, 92].

No significant difference in speed performance was found between PML and NML. However, this result should be interpreted with caution, as the included studies differed substantially in how speed was assessed. Rather than focusing solely on sprint outcomes, where previous research has shown benefits of motivational or self-selected music [85, 93, 94], the dataset al.so included metrics such as time-to-completion in aerobic endurance tests, mean contact time in countermovement jumps, and pedal cadence in submaximal cycling. These measures, while indirectly related to movement velocity, reflect distinct physiological and biomechanical mechanisms. For example, cycling cadence depends not only on rhythm and arousal but also on resistance and pacing strategy. The lack of a clear effect may therefore reflect methodological heterogeneity rather than a true absence of influence. This interpretation aligns with previous endurance research showing that preferred music can extend time to fatigue or improve pacing during time trials [95, 96], although these effects often diminish under high-intensity or prolonged effort conditions where internal fatigue cues dominate perception.

### Effects of music listening on psychological parameters

This meta-analysis found motivation scores were significantly higher under PML compared to NPML and NML. Specifically, compared to NPML and NML, PML reliably elicited higher motivational scores across diverse athletic settings. These findings are in line with previous research showing that self-selected music can significantly enhance intrinsic motivation. For instance, Pettit and Karageorghis [97] observed increased intrinsic motivation in American football players when they trained with self-chosen music. Similarly, Digelidis et al. [98] found that students in physical education classes reported greater lesson satisfaction and intrinsic motivation when allowed to listen to music they selected themselves.

This effect is consistent with the Self-Determination Theory [99], which suggests that intrinsic motivation is enhanced when individuals experience autonomy, competence, and relatedness. Allowing participants to select their own music may therefore provide a greater sense of control over their exercise environment. Prior research supports that preferred or self-selected music can enhance emotional engagement and motivation, both in exercise settings [4] and in broader music contexts [100]. In contrast, NPML may evoke negative affective responses that interfere with enjoyment and diminish motivational drive [101]. Moreover, PML has been associated with making repetitive or demanding exercise more enjoyable and emotionally rewarding [17, 102]. However, while such mechanisms are theoretically supported, empirical confirmation of these psychological pathways remains limited.

In addition to motivational benefits, affective responses were significantly more positive under PML compared to NML (*p* = 0.03). This suggests that individuals enjoy their workouts more when listening to music they like. Music’s impact on emotion has been linked to activation of the brain’s reward system, particularly the mesolimbic dopamine system [1, 103], indicating that emotionally resonant music can produce pleasure-like responses even in physically demanding contexts.

In contrast to its robust effects on motivation and affective response, RPE scores were slightly lower under PML compared to NPML and NML. This relatively small effect may reflect the known limitations of music’s dissociative capacity, particularly at higher intensities, where internal cues such as fatigue and breathlessness dominate attention [92]. Moreover, variability in individual fitness levels and exercise protocols may moderate these effects, as suggested by Mohammadzadeh et al. [104], who found that trained individuals experienced greater reductions in RPE through music than untrained individuals.

### Moderating variables and implications for personalized intervention strategies

The moderator analyses emphasized the differential influence of individual characteristics, notably sex, on psychological responses to preferred music. Specifically, the enhanced motivational (*p* = 0.002) and affective (*p* = 0.018) responses in female-dominant samples align with prior research indicating women’s stronger emotional engagement with music [105, 106]. In contrast, no significant moderating effects were observed for age, music timing, or music choice across any outcomes.

Physically, most performance measures showed minimal sensitivity to individual moderators, supporting previous findings of broadly generalizable ergogenic effects of music [4]. However, a significant moderating effect emerged for sex on strength endurance (*p* = 0.011), with stronger benefits observed in male-dominant studies. This may reflect that submaximal tasks allow greater integration of external auditory cues, facilitating sustained output [107, 108].

When considering the three listening conditions collectively, the results demonstrate a graded response pattern, with PML consistently eliciting the strongest psychological and ergogenic benefits, NPML showing neutral or even slightly adverse effects, and NML serving as a neutral baseline. This gradient likely reflects the interaction of emotional preference, attentional focus, and arousal regulation, with preferred music enhancing affect and motivation, whereas non-preferred tracks may induce affective incongruence or cognitive interference that diminishes these benefits [17, 101].

Thus, while sex appears to modulate the psychological salience of preferred music in women and the endurance-related ergogenic potential in men, the directionality of effects across PML, NPML, and NML underscores that preference alignment is the key determinant of both affective and performance outcomes.

However, it should be noted that the present analyses did not include physiological or neurocognitive parameters associated with central drive, which limits the ability to infer direct causal mechanisms between music exposure and motor performance.

### Strengths and limitations

Overall, the included studies exhibit high methodological quality, and the generally comparable quality levels across studies support internal validity and interpretability of the findings.

Despite the inclusion of 41 studies, key outcome domains such as maximal strength, affective response, and motivation were underrepresented, with many studies involving small samples or lacking full comparisons between music conditions.

The substantial heterogeneity observed across several comparisons likely reflects the limited number of studies per outcome and methodological diversity across studies, including differences in participant characteristics and music conditions that may have introduced implicit confounding factors.Heterogeneity between preferred and no music listening ranged from moderate to high (*I²* = 86–96%; *τ²* = 0.41–5.25), and similarly high heterogeneity was observed in comparisons between preferred and non-preferred music (*I²* = 75–93%; *τ²* = 0.42–12.09). While the applied random-effects model accounts for between-study variance and enhances generalizability, minor data skew may persist due to differences in inclusion criteria, participant profiles, and measurement protocols.

The examination of moderator effects was constrained by inconsistent reporting and non-standardized definitions of music-related variables (e.g., tempo, volume, duration), which complicates comparability across studies. Moreover, the lack of a standardized operationalization of music preference across studies introduces potential selection bias and limits comparability. Small sample sizes and a pronounced sex imbalance (approximately 67% male participants) may further restrict generalizability. Future research should aim to establish clearer and more consistent criteria for music preference determination. Moreover, although the majority of studies focused on physical performance outcomes, the variability in test protocols (e.g., strength vs. cycling tasks) complicates data synthesis and the interpretation of subgroup effects. Additionally, unexplained variability may also stem from unmeasured factors such as participants’ habitual exposure to music, cultural familiarity with musical styles, or testing environments, which were inconsistently reported across studies. However, sensitivity analyses excluding low-quality studies and statistical outliers confirmed the robustness of the findings. Nonetheless, publication bias for several performance outcomes (motivation, strength endurance, aerobic endurance, power output) suggests possible overestimation of these effects, whereas results for other outcomes appear reliable. As an exploratory direction, future research could apply multidimensional similarity approaches (e.g., Euclidean distance measures) to examine how psychological and physical effects of music interrelate.

## Conclusion

This systematic review and meta-analysis indicates that PML is generally more favorable than NPML and NML regarding psychological and physical performance across different exercise contexts. These effects reflect between-condition comparisons at post-test rather than within-condition improvements. Compared to NPML and NML, PML was associated with higher motivation, more positive affective responses, lower RPE, and greater strength endurance, power output, and maximal strength, whereas NPML generally showed neutral effects across psychological parameters. While most physical effects appeared broadly consistent across sex, training level, and task type, psychological outcomes and strength endurance showed sex-related variation, with stronger effects in female-dominant (psychological) and male-dominant (strength) samples. Despite considerable between-study heterogeneity, the overall pattern supports the ergogenic and psychoregulatory potential of PML in sport and exercise. Practitioners and coaches are encouraged to integrate preferred music into training routines as a cost-effective strategy to enhance motivation and physical output. Future research should investigate the underlying mechanisms and long-term effects of PML, and consider individual moderators such as sex to enhance personalized music-based interventions, particularly for psychological outcomes and strength endurance.

## Supplementary Information

Supplementary Material 1.

Supplementary Material 2.

## Data Availability

The data presented in this study are available on request from the corresponding author.
