# Supplementary material for: Effects of preferred music listening on physical and psychological parameters in sports: a systematic review and meta-analysis with meta-regression
Source: BMC Sports Sci Med Rehabil. 2025 Dec 22;18:44. doi: 10.1186/s13102-025-01470-2 (PMC12859869; doi:10.1186/s13102-025-01470-2)
Supplement: Supplementary file 1 — Supplementary Material 1. [file 13102_2025_1470_MOESM1_ESM.docx]

**Table S1** PRISMA Checklist.

| **Section and Topic** | **Item #** | **Checklist item** | **Location where item is reported** |
| --- | --- | --- | --- |
| **TITLE** | | |  |
| Title | 1 | Identify the report as a systematic review. | 1 |
| **ABSTRACT** | | |  |
| Abstract | 2 | See the PRISMA 2020 for Abstracts checklist. | 2 |
| **INTRODUCTION** | | |  |
| Rationale | 3 | Describe the rationale for the review in the context of existing knowledge. | 3 ff. |
| Objectives | 4 | Provide an explicit statement of the objective(s) or question(s) the review addresses. | 4 |
| **METHODS** | | |  |
| Eligibility criteria | 5 | Specify the inclusion and exclusion criteria for the review and how studies were grouped for the syntheses. | 4 |
| Information sources | 6 | Specify all databases, registers, websites, organisations, reference lists and other sources searched or consulted to identify studies. Specify the date when each source was last searched or consulted. | 4 |
| Search strategy | 7 | Present the full search strategies for all databases, registers and websites, including any filters and limits used. | 4 |
| Selection process | 8 | Specify the methods used to decide whether a study met the inclusion criteria of the review, including how many reviewers screened each record and each report retrieved, whether they worked independently, and if applicable, details of automation tools used in the process. | 4 |
| Data collection process | 9 | Specify the methods used to collect data from reports, including how many reviewers collected data from each report, whether they worked independently, any processes for obtaining or confirming data from study investigators, and if applicable, details of automation tools used in the process. | 4 |
| Data items | 10a | List and define all outcomes for which data were sought. Specify whether all results that were compatible with each outcome domain in each study were sought (e.g. for all measures, time points, analyses), and if not, the methods used to decide which results to collect. | 5 ff. |
|  | 10b | List and define all other variables for which data were sought (e.g. participant and intervention characteristics, funding sources). Describe any assumptions made about any missing or unclear information. | 5 ff. |
| Study risk of bias assessment | 11 | Specify the methods used to assess risk of bias in the included studies, including details of the tool(s) used, how many reviewers assessed each study and whether they worked independently, and if applicable, details of automation tools used in the process. | 5 ff. |
| Effect measures | 12 | Specify for each outcome the effect measure(s) (e.g. risk ratio, mean difference) used in the synthesis or presentation of results. | 6 ff. |
| Synthesis methods | 13a | Describe the processes used to decide which studies were eligible for each synthesis (e.g. tabulating the study intervention characteristics and comparing against the planned groups for each synthesis (item #5)). | 5 ff. |
|  | 13b | Describe any methods required to prepare the data for presentation or synthesis, such as handling of missing summary statistics, or data conversions. | 6 ff. |
|  | 13c | Describe any methods used to tabulate or visually display results of individual studies and syntheses. | 6 ff. |
|  | 13d | Describe any methods used to synthesize results and provide a rationale for the choice(s). If meta-analysis was performed, describe the model(s), method(s) to identify the presence and extent of statistical heterogeneity, and software package(s) used. | 6 ff. |
|  | 13e | Describe any methods used to explore possible causes of heterogeneity among study results (e.g. subgroup analysis, meta-regression). | 6 ff. |
|  | 13f | Describe any sensitivity analyses conducted to assess robustness of the synthesized results. | 6 ff. |
| Reporting bias assessment | 14 | Describe any methods used to assess risk of bias due to missing results in a synthesis (arising from reporting biases). | 6 ff. |
| Certainty assessment | 15 | Describe any methods used to assess certainty (or confidence) in the body of evidence for an outcome. | 6 ff. |
| **RESULTS** | | |  |
| Study selection | 16a | Describe the results of the search and selection process, from the number of records identified in the search to the number of studies included in the review, ideally using a flow diagram. | 7 |
|  | 16b | Cite studies that might appear to meet the inclusion criteria, but which were excluded, and explain why they were excluded. | NA |
| Study characteristics | 17 | Cite each included study and present its characteristics. | 9 ff. |
| Risk of bias in studies | 18 | Present assessments of risk of bias for each included study. | 28 ff. / Fig. 10 |
| Results of individual studies | 19 | For all outcomes, present, for each study: (a) summary statistics for each group (where appropriate) and (b) an effect estimate and its precision (e.g. confidence/credible interval), ideally using structured tables or plots. | Table 3 |
| Results of syntheses | 20a | For each synthesis, briefly summarise the characteristics and risk of bias among contributing studies. | 28 ff. |
|  | 20b | Present results of all statistical syntheses conducted. If meta-analysis was done, present for each the summary estimate and its precision (e.g. confidence/credible interval) and measures of statistical heterogeneity. If comparing groups, describe the direction of the effect. | 20 ff. /  Fig. 2-9 |
|  | 20c | Present results of all investigations of possible causes of heterogeneity among study results. | 20 ff. /  Fig. 2-9 |
|  | 20d | Present results of all sensitivity analyses conducted to assess the robustness of the synthesized results. | 29 ff./ Fig. 11-18 |
| Reporting biases | 21 | Present assessments of risk of bias due to missing results (arising from reporting biases) for each synthesis assessed. | 28 ff. / Fig. 10 |
| Certainty of evidence | 22 | Present assessments of certainty (or confidence) in the body of evidence for each outcome assessed. | NA |
| **DISCUSSION** | | |  |
| Discussion | 23a | Provide a general interpretation of the results in the context of other evidence. | 33 ff. |
|  | 23b | Discuss any limitations of the evidence included in the review. | 33 ff. |
|  | 23c | Discuss any limitations of the review processes used. | 33 ff. |
|  | 23d | Discuss implications of the results for practice, policy, and future research. | 36 |
| **OTHER INFORMATION** | | |  |
| Registration and protocol | 24a | Provide registration information for the review, including register name and registration number, or state that the review was not registered. | 2 |
|  | 24b | Indicate where the review protocol can be accessed, or state that a protocol was not prepared. | NA |
|  | 24c | Describe and explain any amendments to information provided at registration or in the protocol. | NA |
| Support | 25 | Describe sources of financial or non-financial support for the review, and the role of the funders or sponsors in the review. | 36 ff. |
| Competing interests | 26 | Declare any competing interests of review authors. | 36 ff. |
| Availability of data, code and other materials | 27 | Report which of the following are publicly available and where they can be found: template data collection forms; data extracted from included studies; data used for all analyses; analytic code; any other materials used in the review. | 36 ff. |

*From:*  Page MJ, McKenzie JE, Bossuyt PM, Boutron I, Hoffmann TC, Mulrow CD, et al. The PRISMA 2020 statement: an updated guideline for reporting systematic reviews. BMJ 2021;372:n71. doi: 10.1136/bmj.n71 For more information, visit: <http://www.prisma-statement.org/>

**Text S1.** Full search terms

**PubMed**

(("preferred music"[Title/Abstract] OR "non-preferred music"[Title/Abstract] OR "self-selected music"[Title/Abstract] OR music[MeSH Terms]) AND ("exercise"[MeSH Terms] OR "sports"[MeSH Terms] OR "athletes"[MeSH Terms] OR "training"[Title/Abstract] OR "physical activity"[Title/Abstract]) AND ("muscle strength"[MeSH Terms] OR "physical endurance"[MeSH Terms] OR "physical performance"[Title/Abstract] OR "motor skills"[MeSH Terms] OR "psychological"[Title/Abstract] OR "motivation"[MeSH Terms] OR "perceived exertion"[Title/Abstract] OR "exertion"[Title/Abstract] OR "affective response"[Title/Abstract] OR "cognition"[MeSH Terms]))

**Embase**

('preferred music':ab,ti OR 'non preferred music':ab,ti OR 'self selected music':ab,ti OR 'music preference'/exp OR 'music listening'/exp) AND ('exercise'/exp OR 'sport'/exp OR athlete*:ab,ti OR training:ab,ti OR 'physical activity'/exp) AND ('strength'/exp OR 'endurance'/exp OR 'power output':ab,ti OR 'performance'/exp OR 'motor control'/exp OR 'psychological'/exp OR motivation:ab,ti OR 'perceived exertion'/exp OR 'affective response':ab,ti OR 'cognition'/exp)

**PsycINFO**

(DE "Music" OR DE "Musical Preference" OR DE "Listening to Music" OR "preferred music" OR "non-preferred music" OR "self-selected music" OR "music preference" OR "music listening") AND

(DE "Exercise" OR DE "Athletes" OR DE "Sports" OR DE "Physical Activity" OR sport OR exercise OR training OR "physical performance" OR "physical activity") AND (DE "Physical Performance" OR DE "Endurance" OR DE "Strength Training" OR DE "Motivation" OR DE "Affect" OR DE "Cognition" OR DE "Fatigue" OR "RPE" OR "rate of perceived exertion" OR "perceived exertion"

OR "psychological outcome" OR "affective response" OR "cognitive performance")

**MEDLINE**

("Music/" OR "Music Therapy/" OR "Listening Behavior/" OR "preferred music".mp. OR "non-preferred music".mp. OR "self-selected music".mp.) AND ("Exercise/" OR "Sports/" OR "Athletes/" OR "Training/" OR "Physical Fitness/" OR sport.mp. OR exercise.mp.) AND ("Muscle Strength/" OR "Physical Endurance/" OR "Performance/" OR "Motor Skills/" OR "Motivation/" OR "Affective Response/" OR "Cognition/" OR "Fatigue/" OR "Perceived Exertion/")

**Table S2** Characteristics of the included studies.

| **References** | **No. of subjects (sex); age (mean ± SD); height (mean ± SD); weight (mean ± SD); population** | **Music conditions; study design** | **Test modality & music modality** | **Outcome measures: Category; Method; Function** | **Results, mean (SD)** | **Level of evidence** |
| --- | --- | --- | --- | --- | --- | --- |
| **Allocca Filho et al.** (2022) | 11 (11 M); 26.64 ± 2.15 yrs; 1.78 ± 0.05 m; 84.15 ± 6.37 kg; CrossFit practitioners | PML (n = 11)  NPML (n = 11)  NML (n = 11); Randomized repeated measures | Warm-up: 6 min running + 30 s each jumping jacks + burpee + mountain climber + squat jumping  Test: 5 sets each “all out” 30 s jumping jacks + burpee + mountain climber + squat jumping; 30 s active rest  Music: during test; free individual choice of music | *Psychological*  Borg scale (0–10); RPE  Brunel Mood Scale (24 items); affective response  *Physical*  Total amount of movements (number); strength endurance | **RPE (score):**  PML: 8.45 (0.93)  NPML: 8.09 (1.44)  NML: 8.72 (1.19)  **Affective response (score):**  PML: 103.90 (6.30)  NPML: 106.36 (9.58)  NML: 103.72 (6.29)  **Strength endurance (number):**  PML: 705.30 (46.32)  NPML: 591.50 (64.47)  NML: 649.10 (41.88) | + |
| **Ballmann et al.** (2019) | 14 (14 M); 20.14 ± 1.79 yrs; 1.80 ± 0.07 m; 78.2 ± 14.09 kg; physically active males | PML (n = 14)  NPML (n = 14); Randomized crossover | Warm-up: 5 min unloaded resistance run  Test: 3x 15 s max. tempo run  Music: during test; free individual choice of music ; 166.9 ± 9.9 bpm | *Psychological*  Borg scale (0–10); RPE  VAS (0–100); motivation  *Physical*  Wingate test (W); power output | **RPE (score):**  PML: 7.2 (0.8)  NPML: 7.7 (0.7)  **Motivation (score):**  PML: 55.8 (10.8)  NPML: 33.9 (21.9)  **Power output (W):**  PML: 787.8 (129.7)  NPML:790.1 (122.7) | + |
| **Ballmann et al.** (2020) | 10 (10 M); 21.6 ± 1.7 yrs; 1.83 ± 0.05 m; 91.56 ± 12.9 kg; resistance-trained males | PML (n = 10)  NPML (n = 10); Randomized crossover | Warm-up: 5 reps (40% 1RM), 3 reps (60% 1RM)  Test: barbell bench press (2.5–20 kg to failure), explosive barbell press (20 kg to failure)  Music: during test ;free individual choice of music; >120bpm | *Psychological*  Borg scale (0–10); RPE  VAS (0–100); motivation *Physical*  RTF (number); strength endurance | **RPE (score):**  PML : 7.79 (1.43)  NPML : 7.75 (1.75)  **Motivation (score):**  PML : 62.2 (30.1)  NPML : 21.5 (19.0)  **Strength endurance (number):**  PML : 9.4 (2.2)  NPML : 8.0 (1.4) | + |
| **Ballmann et al.** (2021b) | 12 (12 M); 20.5 ± 1.2 years; 1.83 ± 0.07 m; 95.1 ± 17.8 kg; strength-trained males | PML (n = 12)  NPML (n = 12); Randomized crossover | Warm-up: barbell bench press (2.5–20 kg to failure), 3x3 explosive barbel lifts (20 kg to failure)  Test: barbell bench press (2.5–20 kg to failure), explosive barbel lift (20 kg to failure)  Music: during test; free individual choice of music; 126.5 ± 26.6 bpm | *Psychological*  VAS (0–100); motivation  *Physical*  RTF (number); strength endurance  1RM (W); power output | **Motivation (score):**  PML: 80.40 (11.20)  NPML: 18.80 (9.29) **Strength endurance (number):**  PML: 10.58 (2.07)  NPML: 8.90 (1.80)  **Power output (W):**  PML: 776.30 (275.70)  NPML:687.60 (229.40) | + |
| **Ballmann et al.** (2021b) | 10 (10 M); 22.8 ± 5.8 yrs; 1.74 ± 0.08 m; 81.0 ± 18.2 kg; resistance-trained males | PML (n = 10)  NML (n = 10); Randomized crossover | Warm-up: barbell bench press 5 reps (50% 1RM) & 3 reps (70% 1RM)  Test: barbell bench press (2.5–20 kg to failure) & 3x explosive presses (20 kg barbell to failure)  &  Warm-up: barbell bench press 5 reps (40% 1RM) & 3 reps (60% 1RM)  Test: explosive barbell bench press 2 reps (75% 1RM) & barbell bench press (75% 1RM to failure)  Music: during test; free individual choice of music; 131 ± 6 bpm | *Psychological*  VAS (0–100); motivation  *Physical*  RTF (number); strength endurance  1RM (W); power output | **Motivation (score):**  PML: 72.40 (20.15)  NML: 52.00 (22.89) **Strength endurance (number):**  PML: 14.30 (2.75)  NML: 12.10 (1.97)  **Power output (W):**  PML: 507.76 (62.84)  NML: 458.35 (61.35) | + |
| **Bartolomei et al.** (2015) | 19 (19 M); 26.6 ± 6.8 yrs; 1.80 ± 0.08 m; 84.7 ± 11.7 kg; students | PML (n = 19)  NML (n = 19); Non-randomized repeated measures | Warm-up: 5min bike ergometer  Test: barbell bench press (70 kg + 2 kg each repetition until failure) & barbell bench press (60% 1RM until failure)  Music: during test; free individual choice of music; >120 bpm | *Physical*  1RM (kg); maximal strength RTF (number); strength endurance | **Maximal strength (kg):**  PML: 103.5 (19.4)  NML: 103.5 (19.9)  **Strength endurance (number):**  PML: 22.52 (0.74)  NML: 21.29 (0.72) | + |
| **Bentouati et al.** (2023) | 10 (10 M); 20.42 ± 1.08 yrs; 1.77 ± 0.05 m; 73.24 ± 11.05 kg; students | PML (n = 10)  NPML (n = 10)  NML (n = 10); Randomized crossover | Warm-up: 10 min cycle ergometer at 60 rpm  Test: 30 s cycle ergometer as fast as possible against a resistance  Music: during warm-up; free individual choice of music; 120–140 bpm | *Psychological*  Borg scale (0–10); RPE  *Physical*  Wingate test (W); power output | **RPE (score):**  PML: 6.00 (0.82)  NPML: 6.60 (0.71)  NML: 7.13 (0.71)  **Power output (W/kg):**  PML: 11.24 (1.12)  NPML: 10.46 (0.75)  NML: 9.29 (1.07) | ++ |
| **Biagini et al.** (2012) | 10 (10 M); 22.3 ± 3.1 yrs; 1.80 ± 0.08 m; 78.4 ± 10.5 kg; recreationally trained male athletes | PML (n = 10)  NML (n = 10); Randomized crossover | Warm-up: 5 min on cycle ergometer at 50 rpm Test: barbell bench press 3 reps (75% 1RM until failure)  Music: during test; free individual choice of music; 80 dB | *Psychological*  Borg scale (6–20); RPE  *Physical*  RTF (number); strength endurance | **RPE (score):**  PML: 8.81 (1.18)  NML: 9.06 (1.09)  **Strength endurance (number):**  PML: 14.15 (1.95)  NML: 13.90 (1.83) | + |
| **Blasco–Lafarga et al.** (2022) | 13 (13 M); 14.85 ± 0.68 yrs; 1.88 ± 0.05 m; 75.89 ± 8.02 kg; young basketball players | PML (n = 13)  NML (n = 13); Randomized repeated measures | Warm-up: 12 minutes  Test: 8 km shuttle run (increase 0.5 km/h every 40 m until exhaustion) & 2x V-cut test (5 x 5 m change of direction run)  Music: during test; the 15 most preferred/non-preferred songs out of 40 given by the researchers; 116.0 ± 17.3 bpm; 90 dB | *Psychological*  Borg scale (0–10); RPE | **RPE (score):**  PML: 6.69 (1.55)  NML: 6.85 (1.63) | + |
| **Chizewski** (2016) | 29 (13 F, 16 M); 22.5 ± 4.5 yrs; 1.74 ± 0.09 m; 73.63 ± 13.67 kg; college students | PML (n = 29)  NML (n= 29); Randomized repeated measures | Warm-up: 3 min jogging on a treadmill  Test: individually paced running on a treadmill for as long as participants wanted (max 1 h)  Testing on separate days  Music: during test; free individual choice of music | *Psychological*  Borg scale (6–20); RPE  PACES (18-126); affective response  *Physical*  Exercise duration (min); strength endurance | **RPE (score):**  PML: 14.37 (2.66)  NML: 13.78 (2.56)  **Affective response (score):**  PML: 102.5 (14.5)  NML: 87.6 (16.5)  **Strength endurance (min):**  PML: 31.04 (14.2)  NML: 25.10 (15.1) | + |
| **Clark et al.** (2021) | 17 (9 F, 8 M); 24.2 ± 4.9 yrs; recreational runners | PML (n = 17)  NML (n = 17); Randomized repeated measures | Warm-up: cycling on a cycle ergometer until target heart rate was reached  Test: 2 x 1.5-mile routes & 1x 3.2-mile route  Music: during test; free individual choice of music; 125.5 bpm | *Pychological*  Borg scale (0–10); RPE | **RPE (score):**  PML: 7.00 (1.19)  NML: 7.5 (0.95) | + |
| **Cole & Maeda** (2015) | 35 (20 F, 15 M); 20.7 ± 2.3 yrs; (F) 1.67 ± 0.07 m; (M) 1.82 ± 0.08 m; undergraduate students | PML (n = 35)  NPML (n = 35)  NML (n = 35); Randomized repeated measures | Warm-up: 10 min jogging and stretching  Test: 12 min Cooper Test  Music: during test; one preferred/non-preferred song chosen for every participant; 137 bpm; 82-85 dB | *Physical*  Total running distance (m); distance | **Aerobic endurance (m):**  PML: 2248 (282)  NPML: 2213 (306)  NML: 2198 (283) | + |
| **de Abreu Araújo et al.** (2018) | 20 (10 F, 10 M); 20.7 ± 1.9 yrs; 1.67 ± 0.1 m; 64.5 ± 10.6 kg; healthy adults | PML (n = 20)  NML (n = 20); repeated-measures | Warm-up: 5 min individually  Test: barbell curl and leg extensor at 80% of 10RM for as many repetitions as possible  Music: during test; free individual choice of music; 121 bpm | *Physical*  RTF (number); strength endurance | **Strength endurance (number):**  PML: 18 (3)  NML: 14 (3) | + |
| **Dos Santos & Carpes** (2021) | 13 (6 F, 7 M); 24 ± 4 yrs;  1.70 ± 0.10 m; 68.3 ± 8.5 kg; physically active adults | PML (n = 13)  NML (n = 13); Randomized crossover | First visit: Bicycle ergometer test  Second visit: Submaximal cycling tests  Third visit: Submaximal cycling tests  Music: during test; free individual choice of music; 129.4 ± 12.2 bpm | *Psychological*  Borg scale (6–20); RPE  *Physical*  Pedal-cadence (rpm); speed | **RPE (score):**  PML: 14 (3)  NML: 13 (3)  **Speed (rpm):**  PML: 85.45 (3.49)  NML: 83.80 (2.97) | + |
| **Greco et al.** (2022) | 26 (26 M); 50.8 ± 8.4 yrs; 1.74 ± 0.07 m; 79.0 ± 9.8 kg; resistance trained males | PML (n = 26)  NML (n = 26); Non-randomized repeated measures | Warm-up: 5 min cycle ergometer at 50 rpm at self-selected load  Test: 5 x maximal isometric contraction held for 3 s + 12 x maximal isometric contraction held for 3 s + 5 min cycle ergometer and stretching  Music: during test; free individual choice of music; 107.4 ± 42.4 bpm | *Psychological*  Borg scale (0–10); RPE  FS (-5–5); affective response  *Physical*  MVIC (N); maximal strength | **RPE (score):**  PML: 3.0 (3.0)  NML: 3.0 (3.3)  **Affective response (score):**  PML: 4.0 (2.0)  NML: 3.0 (1.3)  **Maximal strength (N)**  PML: 547.06 (135.29)  NML: 529.41 (126.47) | **+** |
| **Hammami et al.** (2021) | 18 (18 M); 17.2 ± 0.6 yrs; 1.89 ± 7.0 m; 71.6 ± 6.7 kg; adolecent basketball players | PML (n = 18)  NML (n = 18); Randomized repeated measures | Warm-up: 15 min of running, stretching, calisthenics and preparatory exercises  Test: repeated-change-of-direction sprints: 10 x 20 m (30 s rest)  Music: during test; free individual choice of music; >120 bpm | *Physical*  Peak sprint time (s); speed | **Speed (s):**  PML: 5.75 (0.25)  NML: 5.85 (0.25) | **+** |
| **Hutchinson et al.** (2024) | 47 (19 F, 28 M); 20.6 ± 1.5 yrs; 1.70 ± 0.08 m; 77.9 ± 15.0 kg; college students | PML (n = 47)  NPML (n = 47); Randomized crossover | Warm-up: 30% of 1RM for five reps + 50% of 1RM for three reps  Test: 75% of 1RM for as many repetitions as possible  Music: during test; free individual choice of genre; 119 ± 11 bpm; 75 dB | *Psychological*  Borg scale (6–20); RPE  VAS (0–100); motivation  *Physical*  RTF (number); strength endurance | **RPE (score):**  PML: 15.31 (2.62)  NPML: 15.31 (2.99)  **Motivation (score):**  PML: 67.80 (21.01)  NPML: 56.44 (26.84)  **Strength endurance (number):**  PML: 12.87 (3.10)  NPML: 13.04 (2.91) | + |
| **Jebabli et al.** (2020) | 20 (20 M); 22 ± 1.3 yrs; 1.8 ± 0.3 m; 75.1 ± 7.7 kg; students | PML (n = 20)  NML (n = 20); Randomized repeated measures | Warm-up: 10-minute walking, running and dynamic stretching  Test: 6-min all-out exercise test on a 400 m outdoor track  Music: during test; free individual choice music; 120–140 bpm | *Psychological*  Borg scale (6–20); RPE  *Physical*  Total running distance (m); distance | **RPE (score):**  PML: 17.3 (1.5)  NML: 16.9 (1.3)  **Aerobic endurance (m):**  PML: 1566.55 (179.73)  NML: 1422.35 (178.76) | + |
| **Jebabli et al.** (2022) | 25 (25 M); 21.0 ± 1.1 yrs; 1.80 ± 0.2 m; 71.3 ± 7.1 kg; students | PML (n = 25)  NML (n = 25); Randomized repeated measures | Warm-up: 10-minute jogging, jumping and dynamic stretching  Test: 6-min all-out exercise test on a 400 m outdoor track  Music: during test; free individual choice of music; 130 ± 10 bpm | *Psychological*  Borg scale (6–20); RPE  *Physical*  Total running distance (m); distance  Mean running speed (km/h); speed | **RPE (score):**  PML: 17.3 (1.4)  NML: 17.4 (1.2)  **Aerobic endurance (m):**  PML: 1539.00 (177.43)  NML: 1396.68 (175.38)  **Speed (km/h):**  PML: 15.75 (1.82)  NML: 13.87 (1.85) | + |
| **Jebabli et al.** (2023a) | 19 (19 M); 22.1 ± 1.2 yrs; 1.79 ± 0.06 m; 72.7 ± 9.3 kg; students | PML (n = 19)  NML (n = 19); Randomized repeated measures | Warm-up: 4 min light jogging + lateral displacements + dynamic stretching + jumping  Test: two repeated sprint sets 5 x 20 m, 15 s active recovery  Music: during test; free individual choice of music; >140 bpm; 70 dB | *Psychological*  Borg scale (6–20); RPE  FS (-5–5); affective response  *Physical*  Peak sprint time (s); speed | **RPE (score):**  PML: 15.50 (1.34)  NML: 16.82 (1.53)  **Affective response (score):**  PML: 2.82 (0.80)  NML: 1.68 (1.17)  **Speed (s):**  PML: 3.44 (0.16)  NML: 3.53 (0.13) | + |
| **Jebabli et al.** (2023b) | 24 (24 M); 22.09 ± 1.16 yrs; 1.79 ± 0.13 m; 72.73 ± 9.31 kg; students | PML (n = 24)  NML (n = 24); Randomized repeated measures | Warm-up: 4 min light jogging + lateral displacements, dynamic stretching + jumping  Test: CMJ until exhaustion  Music: during test; free individual choice of music; >140 bpm; 70 dB | *Psychological*  Borg scale (6–20); RPE  FS (-5–5); affective response  *Physical*  RTF (number); strength endurance  Mean contact time (s); speed | **RPE (score):**  PML: 12.06 (2.12)  NML: 11.12 (2.64)  **Affective response (score):**  PML: 2.85 (0.83)  NML: 1.76 (1.45)  **Strength endurance (number):**  PML: 21.72 (8.95)  NML: 19.85 (8.43)  **Speed (s):**  PML: 0.46 (0.08)  NML: 0.48 (0.09) | ++ |
| **Karow et al.** (2020) | 12 (6 F, 6 M); 21.1 ±1.0 yrs; 1.74 ± 0.71 m; 72.5 ± 11.5 kg; physically active adults | PML (n = 12)  NPML (n = 12)  NML (n = 12); Randomized crossover | Warm-up: Rowing machine (50% of max HR)  Test: 2000 m rowing on the rowing machine  Music: during warm-up; free individual choice of music; >120 bpm | *Psychological*  Borg scale (6–20); RPE  VAS (0–100); motivation  *Physical*  Wingate test (W); power output | **RPE (score):**  PML: 14.0 (1.9)  NPML: 13.8 (1.4)  NML: 14.5 (1.2)  **Motivation (score):**  PML: 66 (12)  NPML: 42 (16)  NML: 45 (15)  **Power output (W/kg):**  PML: 2.18 (0.47)  NPML: 2.05 (0.44)  NML: 1.88 (0.61) | ++ |
| **Köse** (2018) | 13 (13 M); 24.69 ± 1.84 yrs; 1.77 ± 0.03 m; 72.69 ± 6.67 kg; students | PML (n = 13)  NML (n = 13); Non-randomized repeated measures | Warm-up: Run of 10 min & 5min static and dynamic training  Test: barbell bench press (maximal weight) & barbell bench press (60% 1RM)  Music: during test; free individual choice of music; 135–140 bpm | *Physical*  1RM (kg); maximal strength RTF (number); strength endurance | **Maximal strength (kg):**  PML: 67.61 (2.57)  NML: 67.44 (2.61)  **Strength endurance (number):**  PML: 22.69 (1.18)  NML: 21.84 (0.98) | + |
| **Köse et al.** (2025) | 16 (16 M); 21.4 ± 2.4 yrs; 1.75 ± 0.47 m; 72.1 ± 2.4 kg; resistance trained males | PML (n = 16)  NPML (n = 16)  NML (n = 16); Randomized crossover | Warm-up: Run of 10 min & 5 min static and dynamic training  Test: bench press (1RM) & lat-pulldown (75% 1RM to failure, 2 sets)  Music: during warm-up and test; free individual choice of music; 137 bpm | *Physical*  1RM (kg); maximal strength  RTF (number); strength endurance  *Psychological*  Borg scale (0–10); RPE | **Maximal strength (kg):**  PML: 88.13 (9.09)  NPML: 88.26 (9.11)  NML: 88.26 (9.13)  **Strength endurance (number):**  PML: 11.40 (0.73)  NPML: 10.93 (0.70)  NML: 10.60 (0.83)  **RPE (score):**  PML: 6.20 (0.56)  NPML: 6.73 (0.59)  NML: 6.86 (0.64) | + |
| **Labudovic et al.** (2024) | 20 (20 F); 30.65 ± 11.29 yrs; 1.69 ± 0.05 m; 65.6 ± 7.8 kg; university students and recreationally active middle-aged women | PML (n = 20)  NML (n = 20); Non-randomized crossover | Warm-up: no specific warm-up  Test: Cardiopulmonary exercise testing until heart rate ≥ 90% on treadmill  Music: during test; free individual choice of music; >120bpm | *Psychological*  Borg scale (6–20); RPE | **RPE (score):**  PML: 18.2 (1.0)  NML: 18.8 (1.0) | + |
| **Latocha et al.** (2024) | 15 (2 F, 23 ± 2 yrs; 1.75 ± 0.01 m; 72 ± 7 kg; 13 M, 22 ± 2 yrs; 1.80 ± 0.06 m; 79.9 ± 10.2 kg); moderately trained people | PML (n = 15)  NML (n = 15); Randomized repeated measures | Warm-up: individually performed by each participant  Test: 3 sets of 3 reps back squats on the smith machine (3 min active rest)  Music: during active rest; free individual choice of music | *Physical*  Back squat relative peak power (W/kg); power output | **Power output (W/kg)**:  PML: 12.44 (3.32)  NML: 12.28 (3.31) | ++ |
| **Marques et al.** (2022) | 16 (16 M); 27.1 ± 3.9 yrs; 1.77 ± 0.05 m; 78.1 ± 9.6 kg; healthy active males | PML (n = 16)  NML (n = 16); Randomized repeated measures | Warm-up: 5 min cycling at 50W  Test: 8 x 15 s sprint interval training on cycle ergometer, 2 min rest between sets  Music: during test; free individual choice of music; >140–160 bpm | *Psychological*  Borg scale (6–20); RPE  FS (-5 - 5); affective response  *Physical*  Wingate test (W/kg); power output | **RPE (score):**  PML: 17.92 (2.05)  NML: 17.42 (2.19)  **Affective response (score):**  PML: 15.13 (2.24)  NML: 15.26 (2.13)  **Power output (W):**  PML: 734 (119)  NML: 765 (113) | + |
| **Meglic et al.** (2021) | 14 (14 F); 19.9 ± 1.3 yrs; 1.74 ± 0.11 m; 67.2 ± 11.1 kg; college soccer and volleyball players | PML (n = 14)  NPML (n = 14); Randomized crossover | Warm-up: 3 min bike ergometer at 50 watts  Test: 3x 15 s max. tempo run  Music: during warm-up; free individual choice of music; >120 bpm | *Psychological*  Borg scale (0–10); RPE  VAS (0–100); motivation  *Physical* Wingate test (W); power output | **RPE (score):**  PML: 5.74 (1.32)  NPML: 5.73 (1.06)  **Motivation (score):**  PML: 37.33 (17.15)  NPML: 16.38 (18.86)  **Power output (W):**  PML: 704.35 (55.43)  NPML: 646.74 (80.43) | + |
| **Nakamura et al.** (2010) | 15 (15 M); 22.8 ± 3.1 yrs; 1.78 ± 0.49 m; 76.9 ± 6.9 kg; recreational cyclists | PML (n = 15)  NPML (n = 15)  NML (n = 15); Non-randomized repeated measures | Test: two trials cycling at 27 km/h until exhaustion  Music: during test; free individual choice of music; 117 ± 29 bpm (PML); 95 ± 28 bpm (NPML) | *Psychological*  Borg scale (6–20); RPE  *Physical*  Total cycling distance (m); distance | **RPE (score):**  PML: 14.0 (2.9)  NPML: 16.0 (2.7)  NML: 15.0 (3.1)  **Aerobic endurance (m):**  PML: 9800 (4600)  NPML: 7100 (3500)  NML: 7700 (3400) | + |
| **Rasteiro et al.** (2020)**¹** | 10 (10 M)  23.0 ± 2.0 yrs; 1.75 ± 0.01 m; 73.3 ± 11.7 kg; recreational runners | PML (n = 10)  NML (n = 10); Randomized crossover | Test: motorized treadmill incremental test  Music: during test; free individual choice of music; 120 ± 24 bpm; 70-85 dB | *Psychological*  Borg scale (0–10); RPE | **RPE (score):**  PML: 12 (1)  NML: 13 (1) | + |
| **Rasteiro et al.** (2020)**²** | 10 (10 F)  20.0 ± 1.0 yrs; 1.65 ± 0.01 m; 59.7 ± 5.3 kg; recreational runners | PML (n = 10)  NML (n = 10); Randomized crossover | Test: motorized treadmill incremental test  Music: during test; free individual choice of music; 122 ± 19 bpm; 70-85 dB | *Psychological*  Borg scale (0–10); RPE | **RPE (score):**  PML: 13 (1)  NML: 13 (2) | + |
| **Ray** (2023) | 25 (25 F); 20.80 ± 1.87 yrs; 1.66 ± 0.07 m; 65.83 ± 9.45 kg; healthy adults | PML (n = 25)  NML (n = 25); Non-randomized repeated measures | Warm-up: 5 min rowing  Test: 15 min rowing task + 5 min cool down  Music: during test; five preferred songs chosen from a pool pop-songs; 130–170 bpm | *Psychological*  Borg scale (6–20); RPE  PACES (18–126); affective response | **RPE (score):**  PML: 10.94 (0.56)  NML: 11.46 (0.45)  **Affective response (score):**  PML: 88.16 (15.52)  NML: 76.05 (15.79) | + |
| **Rhoads et al.** (2021)**¹** | 8 (8 M); 21.6 ± 1.7 yrs; 1.83 ± 0.06 m; 89.3 ± 16.2 kg; active young adults | PML (n = 8)  NML (n = 8); Randomized crossover | Warm-up: 3 min cycling at 50 W  Test: 3 x 15 s Wingate Anaerobic Tests on cycle ergometer  Music: during test; free individual choice of music; >120 bpm | *Psychological*  Borg scale (1–10); RPE  VAS (0–100); motivation  *Physical*  Wingate test (W/kg); power output | **RPE (score):**  PML: 6.67 (1.33)  NML: 6.93 (1.29)  **Motivation (score):**  PML: 66.80 (13.60)  NML: 57.20 (23.20)  **Power output (W/kg):**  PML: 8.67 (1.55)  NML: 8.83 (1.17) | + |
| **Rhoads et al.** (2021)**²** | 8 (8 F); 20.1 ± 1.1 yrs; 1.66 ± 0.05 m; 61.9 ± 6.4 kg; active young adults | PML (n = 8)  NML (n = 8); Randomized crossover | Warm-up: 3 min cycling at 50 W  Test: 3 x 15 s Wingate Anaerobic Tests on cycle ergometer  Music: during test; free individual choice of music; >120 bpm | *Psychological*  Borg scale (1–10); RPE  VAS (0–100); motivation  *Physical*  Wingate test (W/kg); power output | **RPE (score):**  PML: 4.84 (1.20)  NML: 6.12 (0.96)  **Motivation (score):**  PML: 77.56 (13.46)  NML: 53.88 (15.92)  **Power output (W/kg):**  PML: 9.06 (0.77)  NML: 9.78 (1.22) | + |
| **Rogers et al.** (2023) | 12 (12 F); 20.9 ± 0.3 yrs; 1.64 ± 0.05 m; 59.8 ± 8 kgt; physically active females | PML (n = 12)  NPML (n = 12)  NML (n = 12); Randomized crossover | Warm-up: 5min bike ergometer  Test: Explosive 3x counter movement jump & 3x isometric mid-high pull  48 h BV  Music: during test; free individual choice of music; >120 bpm | *Psychological*  VAS (0–100); motivation  *Physical*  MVIC (N); maximal strength CMJ peak power (W); power output | **Motivation (score):**  PML: 75.31 (10.50)  NPML: 41.25 (19.38) NML: 34.06 (21.13)  **Maximal strength (N):**  PML: 1577.19 (268.42)  NPML: 1463.16 (219.30) NML: 1515.79 (192.98)  **Power output (W):**  PML: 2230.00 (310.00)  NPML: 2110.00 (180.00) NML: 2190.00 (330.00) | ++ |
| **Saleh et al.** (2023) | 30 (30 M); 18.27 ± 0.45 yrs; 21.45 ± 1.40 kg/m²; recreational active males | PML (n = 30)  NML (n = 30); Randomized repeated measures | Warm-up: 10 min jogging and stretching  Test: 2.4 km aerobic endurance exercise  Music: during test; free individual choice of music; 121–131 bpm | *Psychological*  Borg scale (1–10); RPE  *Physical*  Time to completion (min); speed | **RPE (score):**  PML: 4.83 (1.15)  NML: 5.43 (0.73)  **Speed (min):**  PML: 11.30 (1.67)  NML: 11.58 (1.59) | + |
| **Silva et al.** (2021) | 20 (20 M); 20 ± 1.4 yrs; 1.71 ± 0.06 m; 70.5 ± 2.8 kg; students | PML (n = 20)  NPML (n = 20)  NML (n = 20); Non-randomized crossover | Test: 3 reps hand grip strength test & barbell bench press (85% 1RM to failure)  Music: during test; free individual choice of genre; 165 ± 5 bpm; 70 dB | *Psychological* Borg scale (0–10); RPE  *Physical*  RTF (number); strength endurance  MVIC (N); maximal strength | **RPE (score):**  PML: 6.01 (1.89)  NPML: 6.42 (1.65)  NML: 6.38 (1.65)  **Strength endurance (number):**  PML: 6.32 (1.50)  NPML: 4.48 (1.22)  NML: 4.42 (1.10)  **Maximal strength (N):**  PML: 494.86 (75.56)  NPML: 450.01 (74.10)  NML: 449.27 (77.02) | ++ |
| **Stork et al.** (2019) | 24 (12 F, 12 M)  24.08 ± 4.61 yrs; 1.73 ± 0.11 m; 69.92 ± 14.95 kg; insufficiently active adults | PML (n = 24)  NML (n = 24); Randomized crossover | Warm-up: 2-3min ergometer First session:  Test: 20 s Wingate Anaerobic Tests against 5% resistance on cycle ergometer  Second to fifth session:  Test: 3x 20 s Wingate Anaerobic Tests against 5% resistance on cycle ergometer  Music: during test; free individual choice of genre; 132-142 bpm; 72 dB | *Psychological*  VAS (0–10); motivation  PACES (18–126); affective response  *Physical*  Wingate test (W); power output | **Motivation (score):**  PML: 8.04 (1.12)  NML: 3.00 (1.74)  **Affective response (score)**:  PML: 89.58 (17.33)  NML: 83.92 (19.49) **Power output (W):**  PML: 456.00 (30.00)  NML: 447.36 (31.04) | ++ |
| **Van den Elzen et al.** (2019) | 153 (96 F, 57 M) >65 yrs; healthy community dwelling people | PML (n = 153):  NPML (n = 153)  NML (n = 153); Non-randomized crossover | Warm-up: one test session with the handheld dynamometer  Test: 3 x maximal handgrip strength test of the dominant hand for each condition  Music: during test; free individual choice of music | *Physical*  MVIC handgrip-strength (N); maximal strength | **Maximal strength (N):**  PML: 284.49 (14.33)  NPML: 274.68 (14.33)  NML: 275.46 (13.53) | - |
| **Tanaka et al.** (2018) | 15 (15 M); 22.9 ± 0.5 yrs; 1.73 ± 0.01 m; 67.5 ± 1.6 kg; recreational active males | PML (n = 15)  NML (n = 15); Non-randomized crossover | Warm-up: 5 min cycling on cycle ergometer at 50 W  Test: 30 min cycling on cycle ergometer at 60% VO2max  Music: during task; free individual choice of genre ; <120 bpm; 80 dB | *Psychological*  Borg scale (6–20); RPE  FAS (1-6); affective response | **RPE (score):**  PML: 14.7 (0.1)  NML: 13.9 (0.1)  **Affective Response (score):**  PML: 4.3 (0.3)  NML: 4.4 (0.3) | + |
| **Zhang et al.** (2023) | 17 (17 M); 21.31 ± 1.01 yrs; 1.79 ± 0.04 m; 74.65 ± 7.05 kg; healthy young men | PML (n = 17)  NML (n = 17); Randomized repeated measures | Warm-up: 10 min on cycle ergometer at 75 rpm with 20% body weight resistance  Test: 30 s Wingate Anaerobic Tests against 7.5% resistance on cycle ergometer  Music: during warm-up; free individual choice of music | *Psychological*  Borg scale (6–20); RPE  *Physical*  Wingate test (W); power output | **RPE (score):**  PML: 17.76 (0.75)  NML: 17.71 (1.21)  **Power output (W):**  PML: 888.20 (170.95)  NML: 825.90 (181.48) | ++ |

*Note.* PML: preferred music listening; NPML: non-preferred music listening; NML: no music listening; bpm: beats per minute; dB: decibel; VAS: visual analogue scale; RPE: rate of perceived exertion; RTF: repetitions to failure; W: watts; PACES: physical activity enjoyment scale; FS: feeling scale; FAS: felt arousal scale; CMJ: counter movement jump; 1RM: one repetition maximum; RPM: revolutions per minute; MVIC: maximum voluntary isometric contraction; Level of evidence: low quality (−), acceptable quality (+), and high quality (++), according to the Scottish Intercollegiate Guidelines Network Methodology checklist.

**Table S4** Scottish Intercollegiate Guidelines Network Methodology checklist for randomized controlled trials.

|  | Ballmann et al. (2020) | Ballmann et al. (2021a) | Ballmann et al. (2021b) | Silva et al. (2021) | Ballmann et al. (2019) | Meglic et al. (2021) | Karow et al. (2020) | Blasco-Lafarga et al. (2022) | Rasteiro et al. (2020) | Clark et al. (2021) | Stork et al. (2019) | Köse (2018) | Bartolomei et al. (2015) | Jebabli et al. (2022) | Jebabli et al. (2020) | Rogers et al. (2023) | Dos Santos & Carpet (2021) | Bentouati et al. (2023) | Zhang et al. (2023) | Jebabli et al. (2023b) | Jebabli et al. (2023a) | Nakamura et al. (2010) | Allocca Filho et al. (2022) | Latocha et al. (2024) |
| --- | --- | --- | --- | --- | --- | --- | --- | --- | --- | --- | --- | --- | --- | --- | --- | --- | --- | --- | --- | --- | --- | --- | --- | --- |
| 1.1 The study addresses an appropriate and clearly focused question. | Yes | Yes | Yes | Yes | Yes | Yes | Yes | Yes | Yes | Yes | Yes | Yes | Yes | Yes | Yes | Yes | Yes | Yes | Yes | Yes | Yes | Yes | Yes | Yes |
| 1.2 The assignment of subjects to treatment groups is randomized. | Yes | Yes | Yes | Yes | Cs | Cs | Yes | Yes | Yes | Yes | Yes | Yes | Yes | Yes | Yes | Yes | Yes | Yes | Yes | Yes | Yes | Yes | Yes | Yes |
| 1.3 An adequate concealment method is used. | No | No | No | No | No | No | No | No | No | No | No | No | No | No | No | No | No | No | No | Yes | No | No | No | No |
| 1.4 The design keeps subjects and investigators ‘blind’ about treatment allocation. | Cs | Cs | Cs | Yes | Cs | Cs | Yes | Cs | No | No | Yes | Cs | No | Cs | Cs | Yes | Cs | Cs | Cs | Yes | No | Cs | No | Yes |
| 1.5 The treatment and control groups are similar at the start of the trial. | Yes | Yes | Yes | Yes | Yes | Yes | Yes | Yes | Yes | Yes | Yes | Yes | Yes | Yes | Yes | Yes | Yes | Yes | Yes | Yes | Yes | Yes | Yes | Yes |
| 1.6 The only difference between groups is the treatment under investigation. | Yes | Yes | Yes | Yes | Yes | Yes | Yes | Yes | No | Yes | Yes | Yes | Yes | Yes | Yes | Yes | Yes | Yes | Yes | Yes | Yes | Yes | Yes | Yes |
| 1.7 All relevant outcomes are measured in a standard, valid and reliable way. | Yes | Yes | Yes | Yes | Yes | Yes | Yes | Yes | Yes | Yes | Yes | Yes | Yes | Yes | Yes | Yes | Yes | Yes | Yes | Yes | Yes | Yes | Yes | Yes |
| 1.8 What percentage of the individuals or clusters recruited into each treatment arm of the study dropped out before the study was completed? | 0% | 0% | 0% | 0% | 0% | 0% | 0% | 27.8% | 0% | 5.9% | 0% | 0% | 5% | 0% | 0% | 0% | 51.9% | 0% | 0% | 0% | 0% | 0% | 0% | 0% |
| 1.9 All the subjects are analyzed in the groups to which they were randomly allocated (often referred to as intention to treat analysis). | Yes | Yes | Yes | Yes | Yes | Yes | Yes | Yes | Yes | Yes | Yes | Yes | Yes | Yes | Yes | Yes | Yes | Yes | Yes | Yes | Yes | Yes | Yes | Yes |
| 1.10 Where the study is carried out at more than one site, results are comparable for all sites. | Cs | Cs | Cs | Na | Cs | Cs | Cs | Na | Na | Na /No | Cs | Cs | Cs | Na /No | Na /No | Cs | Na | Na | Na | Na | Na | Na | Na | Na |
| 2.1 How well was the study done to minimize bias?  Code as follows: High quality (++), Acceptable quality (+), Low quality (-) | + | + | + | ++ | + | + | ++ | + | + | + | ++ | + | + | + | + | ++ | + | ++ | ++ | ++ | + | + | + | ++ |
| 2.2 Taking into account clinical considerations, your evaluation of the methodology used, and the statistical power of the study, are you certain that the overall effect is due to the study intervention? | Yes | Yes | Yes | Yes | Yes | Yes | Yes | Yes | Yes | Yes | Yes | Yes | Yes | Yes | Yes | Yes | Yes | Yes | Yes | Yes | Yes | Yes | Yes | Yes |
| 2.3 Are the results of this study directly applicable to the patient group targeted by this guideline? | Yes | Yes | Yes | Yes | Yes | Yes | Yes | Yes | Yes | Yes | Yes | Yes | Yes | Yes | Yes | Yes | Yes | Yes | Yes | Yes | Yes | Yes | Yes | Yes |
| Response Options: Yes / No / Can`t say (Cs) / Not applicable (Na) | | | | | |  |  |  |  |  |  | | | | | | | | | | | | | |

| Scottish Intercollegiate Guidelines Network Methodology checklist for randomised controlled trials | | | | | | | | | | | | |  |  |  |
| --- | --- | --- | --- | --- | --- | --- | --- | --- | --- | --- | --- | --- | --- | --- | --- |
|  | Cole & Maeda (2015) | Rhoads et al. (2021) | Ray et al. (2021) | Hutchinson et al. (2024) | Saleh et al. (2023) | Greco et al. (2022) | Hammami et al. (2021) | De Abreu Araújo et al. (2018) | Van den Elzen et al. (2019) | Chizewski (2016) | Marques et al. (2021) | Tanaka et al. (2018) | Labudovic et al. (2024) | Biagni et al. (2012) | Köse et al. (2025) |
| 1.1 The study addresses an appropriate and clearly focused question. | Yes | Yes | Yes | Yes | Yes | Yes | Yes | Yes | Yes | Yes | Yes | Yes | Yes | Yes | Yes |
| 1.2 The assignment of subjects to treatment groups is randomized. | Yes | Yes | Cs | Yes | Yes | Yes | Yes | Yes | Yes | Yes | Yes | Yes | Yes | Yes | Yes |
| 1.3 An adequate concealment method is used. | No | No | No | No | No | No | No | No | No | No | No | No | No | No | No |
| 1.4 The design keeps subjects and investigators ‘blind’ about treatment allocation. | Cs | Cs | Cs | Cs | Cs | Cs | Cs | Cs | Cs | No | Cs | No | No | Cs | No |
| 1.5 The treatment and control groups are similar at the start of the trial. | Yes | Yes | Yes | Yes | Yes | Yes | Yes | Yes | Yes | Yes | Yes | Yes | Yes | Yes | Yes |
| 1.6 The only difference between groups is the treatment under investigation. | Yes | Yes | Yes | Yes | Yes | Yes | Yes | Yes | No | Yes | Yes | Yes | Yes | No | Yes |
| 1.7 All relevant outcomes are measured in a standard, valid and reliable way. | Yes | Yes | Yes | Yes | Yes | Yes | Yes | Yes | Yes | Yes | Yes | Yes | Yes | Yes | Yes |
| 1.8 What percentage of the individuals or clusters recruited into each treatment arm of the study dropped out before the study was completed? | 0% | 0% | 13.8% | 0% | 0% | 10.3% | 10% | 0% | 0% | 0% | 5.9% | 0% | 0% | 0% | 0% |
| 1.9 All the subjects are analyzed in the groups to which they were randomly allocated (often referred to as intention to treat analysis). | Yes | Yes | Yes | Yes | Yes | Yes | Yes | Yes | Yes | Yes | Yes | Yes | Yes | Yes | Yes |
| 1.10 Where the study is carried out at more than one site, results are comparable for all sites. | Na | Cs | Cs | Na | Na | Na | Na | Na | Na | Na | Cs | Cs | Na | Na | Na |
| **2.1** How well was the study done to minimize bias?  Code as follows: High quality (++), Acceptable quality (+), Low quality (-) | + | + | + | + | + | + | + | + | - | + | + | + | + | + | + |
| 2.2 Taking into account clinical considerations, your evaluation of the methodology used, and the statistical power of the study, are you certain that the overall effect is due to the study intervention? | Yes | Yes | Yes | Yes | Yes | Yes | Yes | Yes | Yes | Yes | Yes | Yes | Yes | Yes | Yes |
| 2.3 Are the results of this study directly applicable to the patient group targeted by this guideline? | Yes | Yes | Yes | Yes | Yes | Yes | Yes | Yes | Yes | Yes | Yes | Yes | Yes | Yes | Yes |
| Response Options: Yes / No / Can`t say (Cs) / Not applicable (Na) | | | | | |  |  |  |  |  |  |  |  |  |  |

**Table S5.** Results of meta regression analyses.

| **Outcome/Moderator** |  | | | | | | |
| --- | --- | --- | --- | --- | --- | --- | --- |
| **RPE** | **k** | ***ß*** | ***p*** | ***τ²*** | ***I²* (%)** | **R² (%)** | **SE(τ²)** |
| Age | 36 | -0.0129 | 0.48 | 0.2535 | 78.37 | 0.00 | 0.0812 |
| Sex | 36 | 0.0937 | 0.75 | 0.2578 | 78.92 | 0.00 | 0.0820 |
| Music choice | 36 | -0.0558 | 0.83 | 0.2611 | 78.90 | 0.00 | 0.0829 |
| Music timing | 36 | 0.0960 | 0.74 | 0.2580 | 79.04 | 0.00 | 0.0820 |
| **Motivation** |  |  |  |  |  |  |  |
| Age | 13 | -0.5873 | 0.49 | 7.0928 | 88.18 | 0.00 | 3.8633 |
| Sex | 13 | 1.2121 | 0.002 | 6.6275 | 87.10 | 0.00 | 3.8166 |
| Music choice | 13 | -3.1218 | 0.18 | 5.0268 | 82.72 | 18.72 | 2.9415 |
| Music timing | 13 | -0.3453 | 0.88 | 7.5509 | 90.17 | 0.00 | 4.0390 |
| **Affective Response** |  |  |  |  |  |  |  |
| Age | 9 | -0.0351 | 0.52 | 2.0072 | 95.53 | 0.00 | 1.0618 |
| Sex | 9 | 3.0607 | 0.02 | 0.7394 | 89.79 | 59.81 | 0.4192 |
| Music choice | 9 | 0.2771 | 0.79 | 2.0208 | 95.81 | 0.00 | 1.0655 |
| Music timing | 9 | NA | NA | NA | NA | NA | NA |
| **Strength Endurance** |  |  |  |  |  |  |  |
| Age | 16 | 0.2491 | 0.25 | 0.8428 | 89.44 | 0.00 | 0.3857 |
| Sex | 16 | 1.2224 | 0.01 | 0.5719 | 83.88 | 0.00 | 0.2768 |
| Music choice | 16 | -0.4949 | 0.51 | 0.5244 | 83.39 | 0.00 | 0.2570 |
| Music timing | 16 | NA | NA | NA | NA | NA | NA |
| **Power Output** |  |  |  |  |  |  |  |
| Age | 16 | -0.5767 | 0.17 | 7.3205 | 97.67 | 7.84 | 2.9774 |
| Sex | 16 | 1.7376 | 0.37 | 0.4045 | 89.01 | 2.44 | 0.3231 |
| Music choice | 16 | -0.4069 | 0.90 | 8.6848 | 97.95 | 0.00 | 3.5055 |
| Music timing | 16 | 0.6497 | 0.69 | 8.4868 | 97.93 | 0.00 | 3.4203 |
| **Maximal Strength** |  |  |  |  |  |  |  |
| Age | 11 | -0.0134 | 0.77 | 5.9866 | 98.57 | 0.00 | 3.0095 |
| Sex | 11 | 3.1939 | 0.09 | 4.0066 | 97.89 | 23.70 | 2.0412 |
| Music choice | 11 | 0.0840 | 0.96 | 5.9678 | 98.66 | 0.00 | 2.9919 |
| Music timing | 11 | NA | NA | NA | NA | NA | NA |
| **Aerobic Endurance** |  |  |  |  |  |  |  |
| Age | 6 | 13.8264 | 0.09 | 35.5671 | 98.75 | 84.55 | 30.5898 |
| Sex | 6 | -34.979 | 0.15 | 152.4224 | 99.70 | 33.77 | 119.0394 |
| Music choice | 6 | -19.9689 | 0.15 | 152.4224 | 99.70 | 33.77 | 119.0394 |
| Music timing | 6 | NA | NA | NA | NA | NA | NA |
| **Speed** |  |  |  |  |  |  |  |
| Age | 6 | 0.1221 | 0.37 | 0.4187 | 88.14 | 0.00 | 0.3386 |
| Sex | 6 | -0.8028 | 0.37 | 0.4045 | 89.01 | 2.44 | 0.3231 |
| Music choice | 6 | NA | NA | NA | NA | NA | NA |
| Music timing | 6 | NA | NA | NA | NA | NA | NA |

Note. k = number of included studies; τ² = between-study variance; I² = heterogeneity;
R² = explained variance; SE(τ²) = standard error of the between-study variance estimate;
NA = insufficient data or study count.
