# Supplementary material for: Effects of preferred music listening on physical and psychological parameters in sports: a systematic review and meta-analysis with meta-regression
Source: BMC Sports Sci Med Rehabil. 2025 Dec 22;18:44. doi: 10.1186/s13102-025-01470-2 (PMC12859869; doi:10.1186/s13102-025-01470-2)
Supplement: Supplementary file 2 — Supplementary Material 2. [file 13102_2025_1470_MOESM2_ESM.docx]

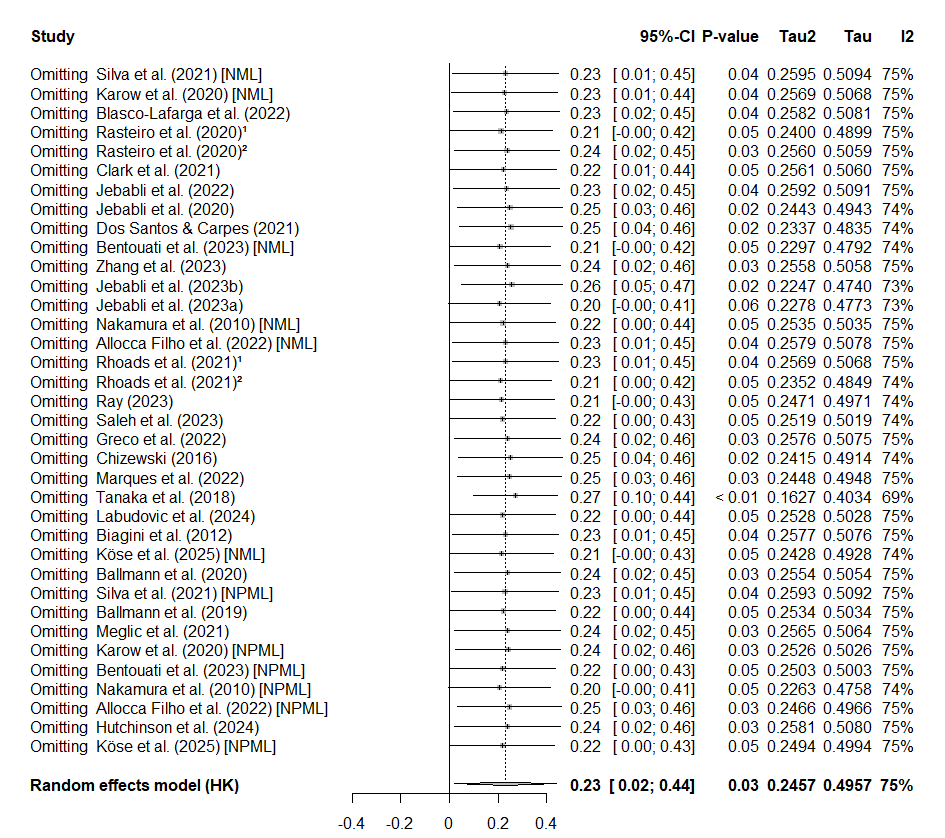


**Figure S1.** L1O analysis: Effect of study omission on RPE

Note. *CI*: confidence interval; *NML*: no music listening; *PML*: preferred music listening; *I²*: heterogeneity.


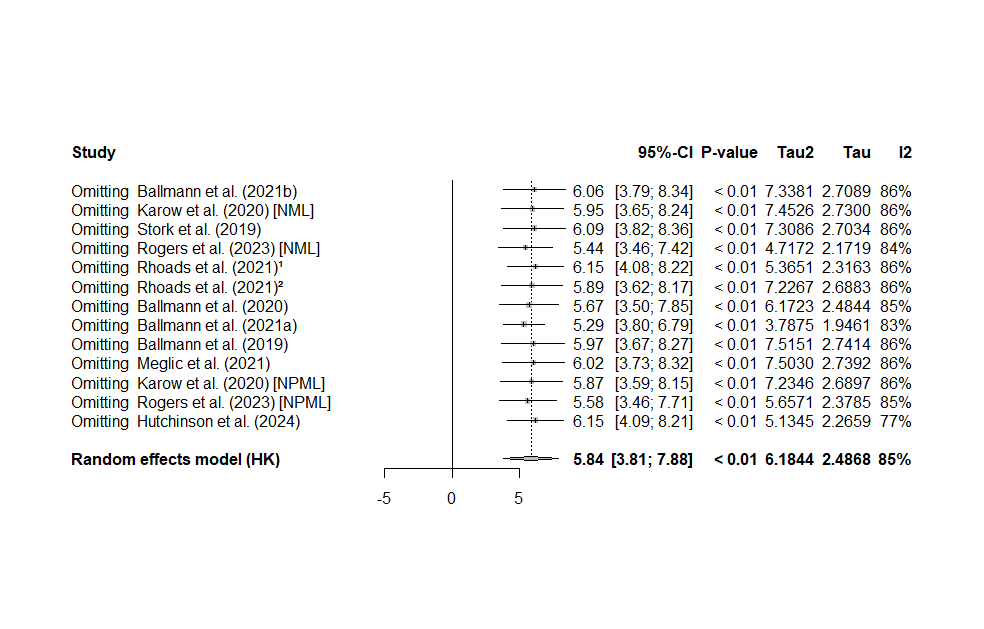


**Figure S2.** L1O analysis: Effect of study omission on motivation

Note. *CI*: confidence interval; *NML*: no music listening; *PML*: preferred music listening; *I²*: heterogeneity.


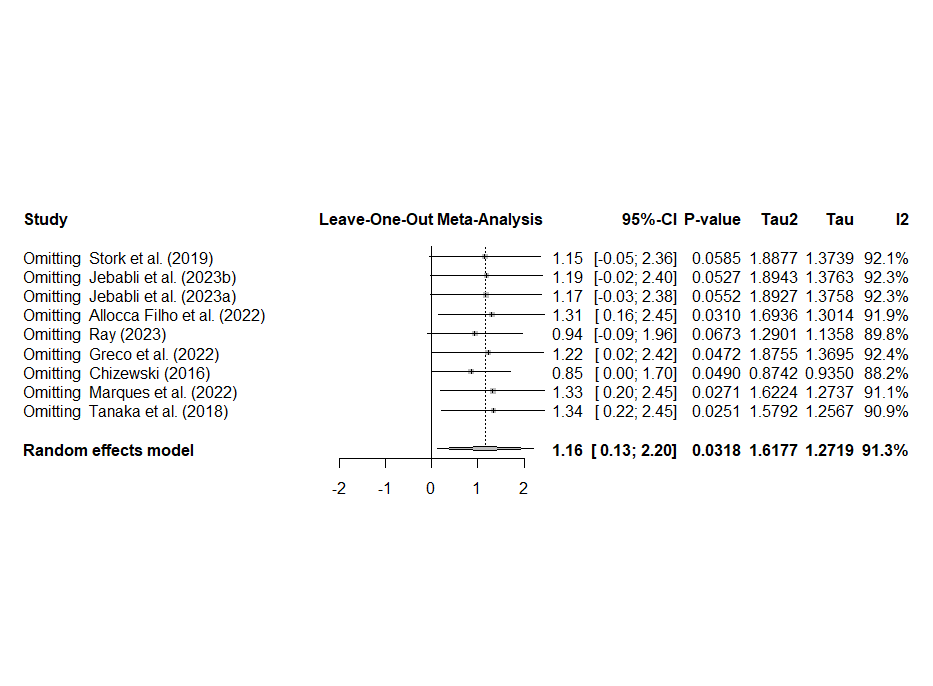


**Figure S3.** L1O analysis: Effect of study omission on affective response

Note. *CI*: confidence interval; *NML*: no music listening; *PML*: preferred music listening; *I²*: heterogeneity.


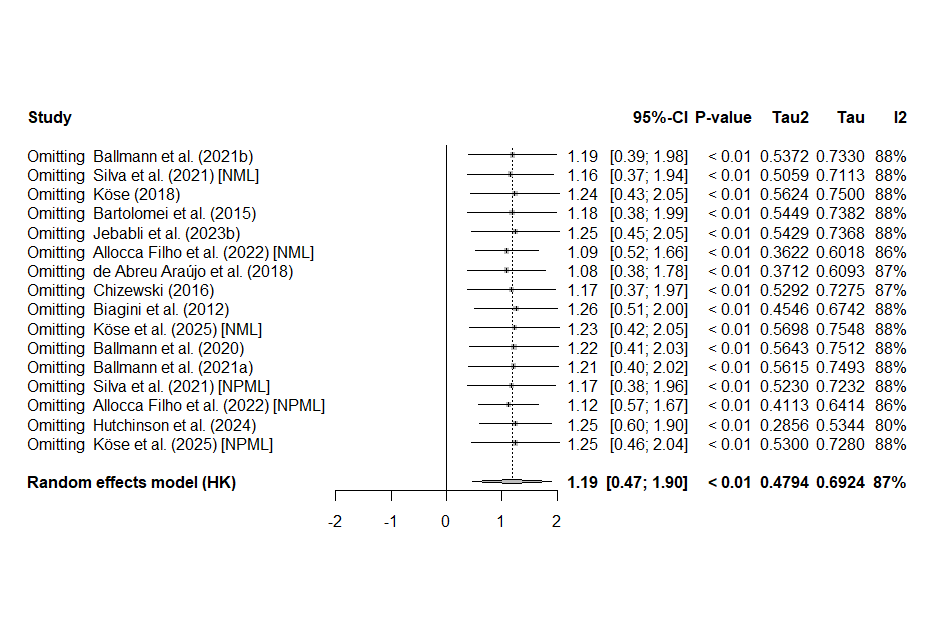


**Figure S4.** L1O analysis: Effect of study omission on strength endurance

Note. *CI*: confidence interval; *NML*: no music listening; *PML*: preferred music listening; *I²*: heterogeneity.


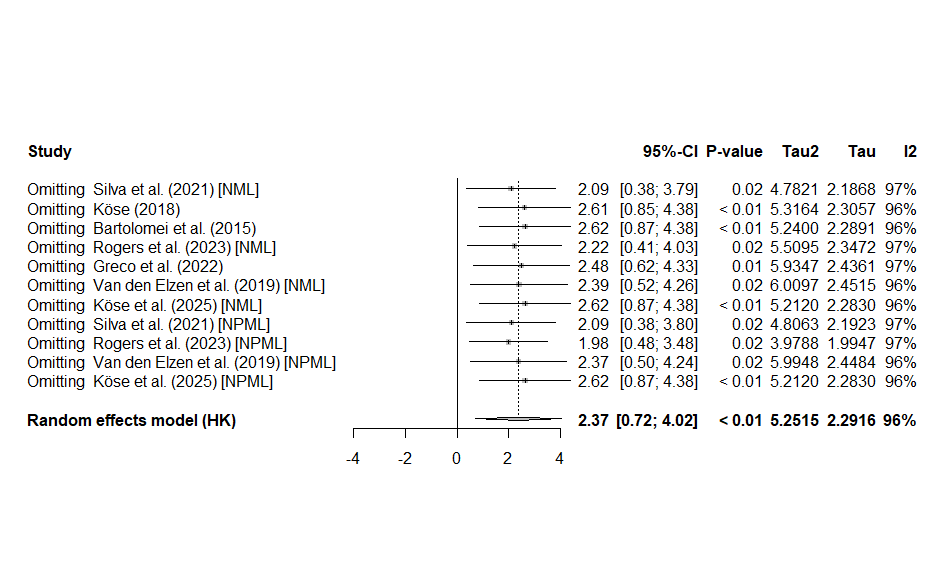


**Figure S5.** L1O analysis: Effect of study omission on maximal strength

Note. *CI*: confidence interval; *NML*: no music listening; *PML*: preferred music listening; *I²*: heterogeneity.


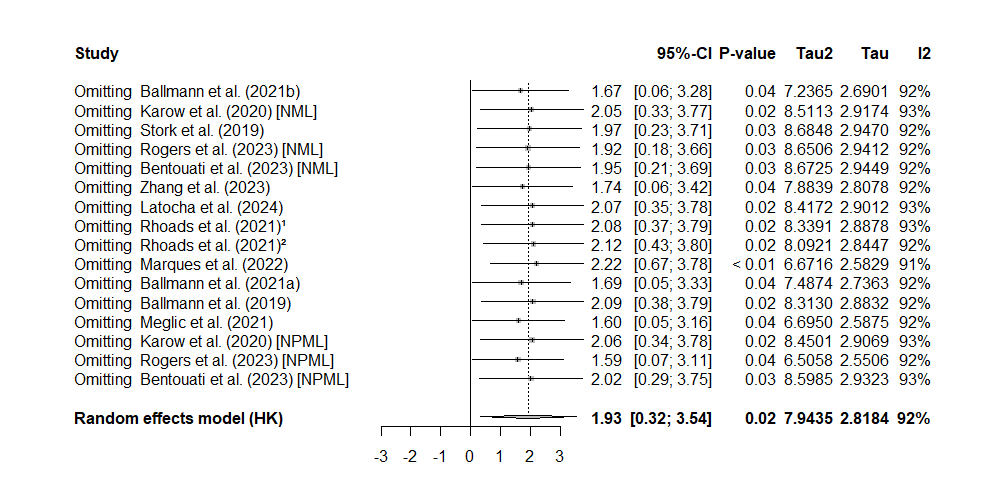


**Figure S6.** L1O analysis: Effect of study omission on power output

Note. *CI*: confidence interval; *NML*: no music listening; *PML*: preferred music listening; *I²*: heterogeneity.


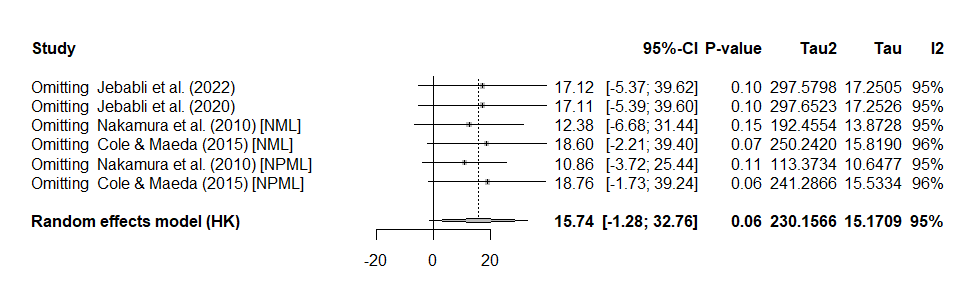


**Figure S7.** L1O analysis: Effect of study omission on aerobic endurance

Note. *CI*: confidence interval; *NML*: no music listening; *PML*: preferred music listening; *I²*: heterogeneity.


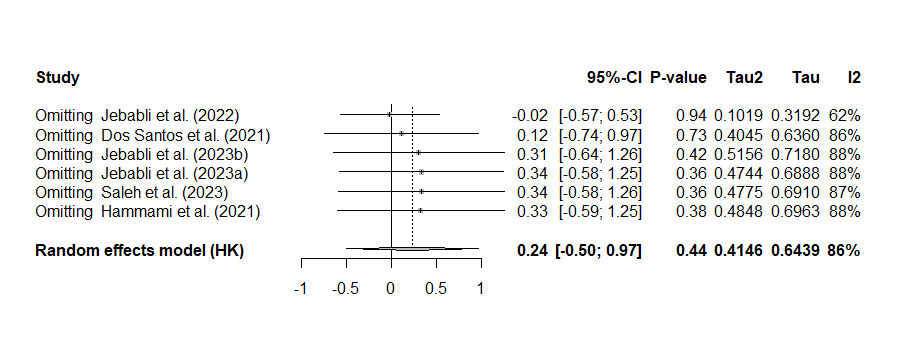


**Figure S8.** L1O analysis: Effect of study omission on speed

Note. *CI*: confidence interval; *NML*: no music listening; *PML*: preferred music listening; *I²*: heterogeneity.
